# Molecular basis for ubiquitin/Fubi cross-reactivity in USP16 and USP36

**DOI:** 10.1038/s41589-023-01388-1

**Published:** 2023-07-13

**Authors:** Rachel O’Dea, Nafizul Kazi, Alicia Hoffmann-Benito, Zhou Zhao, Sarah Recknagel, Kim Wendrich, Petra Janning, Malte Gersch

**Affiliations:** 1 Chemical Genomics Centre, Max Planck Institute of Molecular Physiology, Dortmund, Germany; 2 Department of Chemistry and Chemical Biology, TU Dortmund University, Dortmund, Germany; 3 Department of Chemical Biology, Max Planck Institute of Molecular Physiology, Dortmund, Germany

**Keywords:** X-ray crystallography, Proteins, Post-translational modifications

## Abstract

Ubiquitin and ubiquitin-like proteins typically use distinct machineries to facilitate diverse functions. The immunosuppressive ubiquitin-like protein Fubi is synthesized as an N-terminal fusion to a ribosomal protein (Fubi-S30). Its proteolytic maturation by the nucleolar deubiquitinase USP36 is strictly required for translationally competent ribosomes. What endows USP36 with this activity, how Fubi is recognized and whether other Fubi proteases exist are unclear. Here, we report a chemical tool kit that facilitated the discovery of dual ubiquitin/Fubi cleavage activity in USP16 in addition to USP36 by chemoproteomics. Crystal structures of USP36 complexed with Fubi and ubiquitin uncover its substrate recognition mechanism and explain how other deubiquitinases are restricted from Fubi. Furthermore, we introduce Fubi C-terminal hydrolase measurements and reveal a synergistic role of USP16 in Fubi-S30 maturation. Our data highlight how ubiquitin/Fubi specificity is achieved in a subset of human deubiquitinases and open the door to a systematic investigation of the Fubi system.

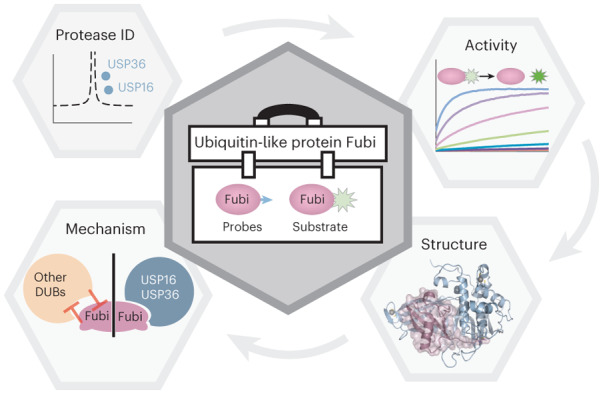

## Abstract (web summary)

A probe for the ubiquitin-like protein Fubi led to the discovery of dual ubiquitin/Fubi C-terminal hydrolase activity in the deubiquitinase USP16 in addition to USP36, enabling structural characterization of this distinctive Ub/Ubl specificity, and revealed a synergistic role of USP16 in ribosomal protein maturation.

## Main

**Fig. 1:**
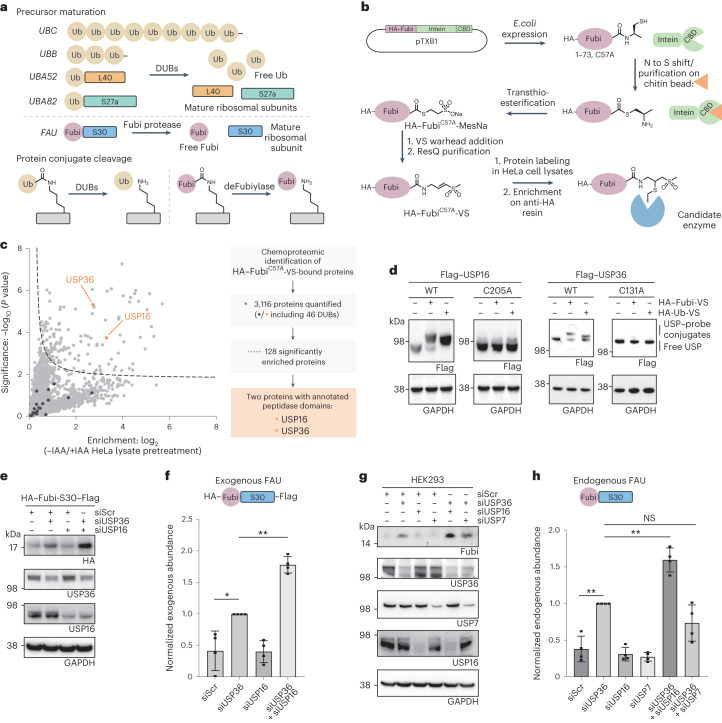
Chemoproteomic identification of USP16 and USP36 as Fubi probe-cross-reactive DUBs that cleave Fubi-S30 in cells. **a**, Schematic representation of the roles of DUBs and Fubi protease/deFubiylase enzymes in protein precursor maturation (top) and processing of protein conjugates (bottom). Names of genes are given in italics; Ub, ubiquitin. **b**, Workflow of Fubi probe semisynthesis for Fubi protease trapping. C-terminally functionalized HA–Fubi species were obtained through expressed protein ligation and native purification. Candidate enzymes were trapped in lysates and enriched on beads, followed by identification by MS; CBD, chitin-binding domain. **c**, Chemoproteomic identification of HA–Fubi^C57A^-VS-bound proteins from HeLa cell lysates. The volcano plot shows the relative label-free abundance ratio (fold change) of proteins detected in samples with or without cysteine alkylation through IAA pretreatment. USP16 and USP36 as the only hits with annotated peptidase domains are highlighted. Other significantly enriched proteins include enzymes with hyperreactive cysteines. **d**, Assessment of USP16 and USP36 probe reactivity. Flag-tagged full-length USP16 and USP36 were overexpressed in HEK293 cells as wild-type (WT) or catalytic cysteine mutants. Lysates were incubated with the indicated probes for 1 h at 37 °C, and reactivity was assessed by western blotting. **e**, Assessment of HA–Fubi-S30–Flag accumulation after depletion of USP16 and USP36. Proteins were depleted in HEK293 cells for 24 h, followed by overexpression of HA–Fubi-S30–Flag for another 24 h; siScr, scrambled control siRNA. **f**, Quantification of HA–Fubi-S30–Flag intensity in samples described in **e**; FAU, Fubi-S30. Values shown correspond to the mean intensities of HA bands of four biologically independent experiments; standard deviations are indicated. Statistical significance was analyzed using individual one-sample, two-tailed *t*-tests comparing to the hypothetical mean of 1 as set for siUSP36 samples; *P ≤ 0.05; **P ≤ 0.01. Additional details on the analysis are given in Statistics and reproducibility. **g**, Assessment of endogenous Fubi-S30 accumulation after USP36 and USP16 depletion in HEK293 cells after 48 h; USP7 was depleted as a control. **h**, Quantification of Fubi-S30 intensity in samples described in **g**. Values shown correspond to the mean of four biologically independent experiments; standard deviations are indicated. Statistical significance was analyzed as in **f**; **P ≤ 0.01; NS, not significant. Source data

Post-translational modifications with ubiquitin regulate numerous cellular processes through degradative and non-degradative signaling^1^. In higher eukaryotes, the 76-amino-acid-long small protein ubiquitin is encoded by four genes, of which *UBA52* and *UBA82* encode ubiquitin as N-terminal fusions to ribosomal proteins L40 and S27a (Fig. 1a)^2^. Cleavage of these fusion proteins through the protease activity of deubiquitinases (DUBs) is essential for the assembly of both ribosomal subunits^2–5^, which elegantly couples cellular protein synthesis to protein degradation capacity.

The ubiquitin system acts in parallel to various systems of ubiquitin-like (Ubl) modifiers, which include NEDD8, ISG15, FAT10, UFM1 and SUMO homologs^6–8^. These Ubl modifiers share the β-grasp fold of ubiquitin, but typically distinct machineries exist for their attachment to substrates and for cleavage of conjugates. How writers, readers and erasers achieve specificity for Ubl modifiers and how these facilitate interactions distinct from ubiquitination are actively being studied. Cross-reactivity toward ubiquitin and a Ubl modifier has been investigated^9,10^, including for PLpro proteases of coronaviruses, which show structural homology to the ubiquitin-specific protease (USP) family of human DUBs^11,12^ and cleave ISG15 and Lys 48-linked polyubiquitin^13,14^. Moreover, the zebrafish ortholog of USP18, which in humans displays exquisite specificity for ISG15, shows cross-reactivity to ubiquitin^15^. Recent work on the dual-specific E1-activating enzyme UBA6 revealed how both ubiquitin and FAT10 are recognized^16,17^.

One particularly poorly studied Ubl modifier is the 74-amino-acid-long protein Fubi^18^, which, like ubiquitin, is synthesized as an N-terminal fusion to a ribosomal protein (S30)^19,20^ from the *FAU* gene and is conserved down to single-cell eukaryotes^21^. S30 is positioned near the A-site decoding center of the small ribosomal subunit, and its maturation through cleavage of Fubi-S30 is essential for translational competence^22^. Fubi shares 36% sequence identity and 61% sequence similarity with ubiquitin and also features a C-terminal diglycine motif. Its conjugation through isopeptide linkages (‘Fubiylation’) has been observed for a few substrates, including T cell antigen receptor-α (TCRα)^23^, BCL-G^24^, endophilin II^25^ and 10-formyltetrahydrofolate dehydrogenase^26^, in line with roles in immunomodulation, embryo implantation^27^ and apoptosis^28^. Similar to ISG15, some Fubi conjugates are secreted and recognized by receptors of the interleukin family^29,30^. Immunosuppressive functions of these conjugates, which are formed in CD8^+^ T cells after stimulation by interferon-γ, have been specifically traced to Fubi^31^. Fubi suppresses immunoglobulin secretion from lymphocytes, inhibits the production of tumor necrosis factor-α by lipopolysaccharide-activated macrophages and reduces the proliferation of mitogen-activated B and T cells^32–34^.

Despite these important roles, the Fubi system is severely understudied on the molecular level compared to other protein conjugate-forming Ubl modifiers. Chemical tools that would facilitate the discovery and characterization of new components are lacking, and how writers, readers and erasers bind Fubi is not known. Recently, the Kutay lab reported the identification of the nucleolar DUB USP36 from ribosome pulldown assays as the first Fubi-S30-cleaving protease^22^. USP36 is the only catalytically active DUB localized to the nucleolus^35^, and it controls its structure through its catalytic activity^36,37^. However, what seemingly restricts the majority of DUBs from cleaving Fubi-S30 and whether there exists redundancy for this function essential for the biogenesis of the small ribosomal subunit is unclear. Importantly, how Fubi is specifically recognized and distinguished from ubiquitin and what endows USP36 and prospective other deFubiylases with the ability to act on Fubi is not known.

Here, we report a chemical tool kit for this understudied Ubl protein, which led to the discovery of dual ubiquitin/Fubi cleavage activity in the DUB USP16 in addition to USP36 through chemoproteomics. We quantify Fubi C-terminal hydrolase activity and reveal a synergistic role of USP16 in S30 maturation. Structures of USP36 in complex with Fubi and ubiquitin probes enabled the identification and validation of evolutionarily conserved interfaces unique to Fubi and both of the identified USPs. Collectively, our data show on the molecular level how ubiquitin/Ubl modifier cross-reactivity, essential for translational competence, is achieved in a subset of human DUBs and open the door to the systematic investigation of Fubiylation.

## Results

### Chemoproteomic identification of Fubi probe-reactive DUBs

**Extended Data Fig. 1:**
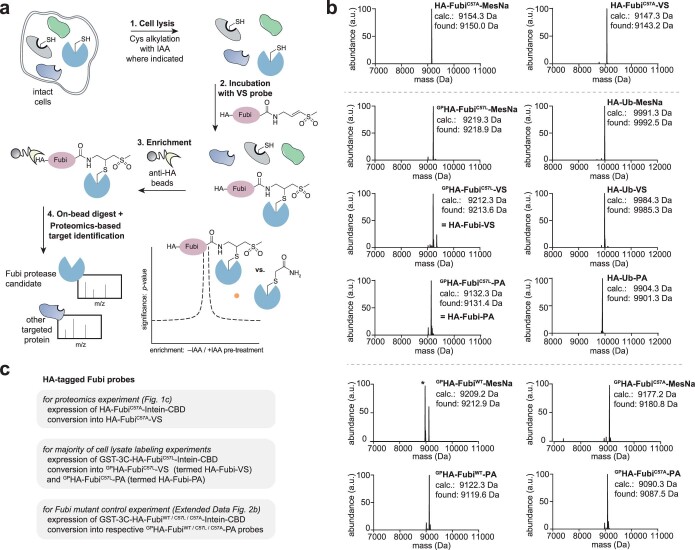
Characterization of Fubi-based probes. **a**. Schematic of the chemoproteomics experimental workflow used to identify Fubi proteases. **b**. Characterization of indicated samples by intact protein mass spectrometry. HA-Fubi-MesNa and derived proteins featured Cys57 mutations to suppress reaction of the thiol with the VS warhead during probe assembly. Analogously mutated and wild-type HA-Fubi-PA probes were prepared for comparison. *, species with a C-terminal carboxylate which was separated during purification. **c**. Overview of different versions of HA-tagged Fubi probes used in this work. Source data

**Extended Data Fig. 2:**
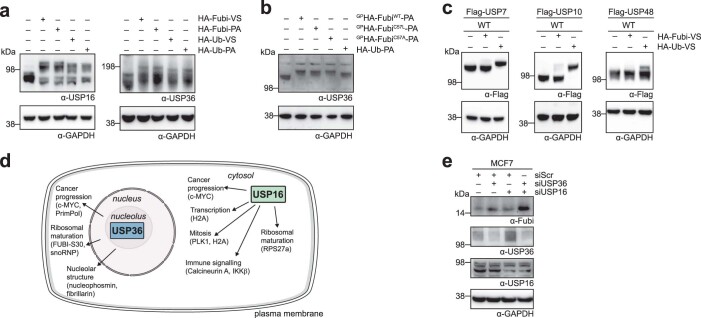
Reactivity of USP DUBs in HEK293 lysate. **a**. Assessment of endogenous USP16 and USP36 reactivity towards Ubiquitin and Fubi probes. HEK293 lysate was incubated with indicated probes (1 h, 37 °C) and reactivity was assessed by Western blot. Uncropped blots are provided as Source Data. **b**. Cellular labeling experiment as in **a** with indicated HA-Fubi-PA probes, demonstrating that mutation of Cys57 in Fubi does not affect reactivity toward USP36. **c**. Assessment of additional USP probe reactivity. Flag-tagged, full-length USP7, USP10 and USP48 were overexpressed in HEK293 and analyzed as in Fig. 1d. **d**. Depiction of cellular roles of USP16 and USP36. Processes to which USP16 and USP36 activity has been connected are listed, with substrates in parentheses. **e**. Assessment of endogenous Fubi-S30 (FAU) accumulation upon USP36 and USP16 depletion in MCF7 cells after 48 h. Compare to Fig. 1g for HEK293 cells. Source data

We reasoned that an activity-based chemoproteomic workflow would provide a comprehensive set of candidate enzymes with Fubi C-terminal hydrolase activity (Extended Data Fig. 1a). Building on the observation that cleavage activity of lysates on a Fubi-TCRα conjugate purified from mouse splenocytes (also known as monoclonal non-specific suppressor factor) is completely abrogated by cysteine modification through Ellman’s reagent^23^, we speculated that deFubiylase enzymes are members of the cysteine hydrolase superfamily. Vinyl sulfones (VSs) are widely used in probes for cysteine-dependent enzymes^38^, and ubiquitin probes equipped with a VS warhead provide broad coverage of cysteine-dependent DUBs from various families^39–41^. We therefore devised semisynthetic access to a hemagglutinin (HA)–Fubi-VS probe in which the N-terminal HA tag serves as an enrichment handle, Fubi serves as a recognition element, and a C-terminal VS warhead serves as a covalent trap for active site cysteines of Fubi-recognizing enzymes (Fig. 1b). To suppress probe multimerization, we used a C57A Fubi variant and obtained low amounts of an HA–Fubi–2-mercaptoethanesulfonic acid (HA–Fubi–MesNa) thioester through bacterial expression of an intein fusion^42^. C-terminal functionalization with (*E*)-3-(methylsulfonyl)prop-2-*en*-1-amine and native purification via anion exchange chromatography furnished HA–Fubi^C57A^-VS (Fig. 1b and Extended Data Fig. 1b,c). Extensive optimization of a workflow for ubiquitin probes concluded that baseline reactivity of VS warheads is best accounted for by comparing probes with the same warhead and different recognition elements^41^. However, because we could not exclude cross-reactivity of prospective deFubiylases with ubiquitin or other Ubl modifiers, we proceeded with comparing HA–Fubi^C57A^-VS-reactive proteins from HeLa cell lysates with or without cysteine alkylation through iodoacetamide (IAA) pretreatment (Extended Data Fig. 1a). Filtering of enriched proteins yielded only two proteins with an annotated peptidase domain: USP16 and USP36 (Fig. 1c). To validate these hits, we first devised access to larger amounts of probe through inclusion of a cleavable glutathione *S*-transferase (GST) tag and a C57L mutation (Extended Data Fig. 1b,c). We next overexpressed full-length USP16 and USP36 in HEK293 cells and assessed their ability to react with either HA–Fubi-VS or HA–ubiquitin-VS by western blotting. Covalent USP–probe conjugates were observed for both enzymes with both probes in a catalytic cysteine-dependent manner (Fig. 1d). Likewise, endogenous USP16 and USP36 reacted with both Fubi and ubiquitin probes featuring the VS and less reactive propargylamine (PA)^43^ warheads (Extended Data Fig. 2a,b), substantiating the chemoproteomic findings and suggesting specific recognition of Fubi by these two DUBs. Other USP DUBs, including USP7, USP10 and USP48, which were detected but not enriched in the proteomics experiment, reacted only with the ubiquitin probe (Extended Data Fig. 2c). Collectively, these results suggest that both USP16 and USP36 feature dual ubiquitin and Fubi C-terminal hydrolase activity.

### A two-tier system of Fubi-S30 cleavage for ribosomal maturation

With various cellular roles reported for both USP16 (refs. ^44–46^) and USP36 (refs. ^22,36,37,47,48^; Extended Data Fig. 2d), we next wanted to assess if both enzymes possess Fubi cleavage activity in cells and in a complementary manner. We noted that USP36 is typically localized to the nucleolus^36^, whereas USP16 is maintained in the cytosol^44^ except during mitosis, suggesting that these DUBs may contribute Fubi processing activity at distinct subcellular localizations and in a two-tier manner. The role of USP36 in cleaving the Fubi-S30 precursor was recently reported^22^, and USP16 was also reported to contribute to ribosomal biogenesis by removing a monoubiquitination of RPS27a in the late cytoplasmic stages of 40S maturation^45^; however, its role in Fubi-S30 cleavage had not been investigated. We therefore overexpressed a tagged Fubi-S30 fusion in HEK293 cells and quantified the accumulation of uncleaved precursor in cells depleted of either DUB alone or in combination by short interfering RNA (siRNA; Fig. 1e,f). Depletion of USP36 led to an increase in the precursor, consistent with recently reported data^22^, whereas depletion of USP16 did not. However, co-depletion of USP16 and USP36 showed a strong increase in precursor levels compared to USP36 depletion alone. The same pattern was consistently observed when cleavage of endogenous Fubi-S30 was assessed (Fig. 1g,h), using the Fubi probe-nonreactive DUB USP7 as a control. Moreover, the same effect on endogenous Fubi-S30 levels was observed in MCF7 cells (Extended Data Fig. 2e). These data confirm cellular Fubi protease activity of both DUBs and are consistent with synergistic roles of these enzymes during biogenesis of the small ribosomal subunit.

### Ubiquitin and Fubi C-terminal hydrolase activity in USP deubiquitinases

**Fig. 2:**
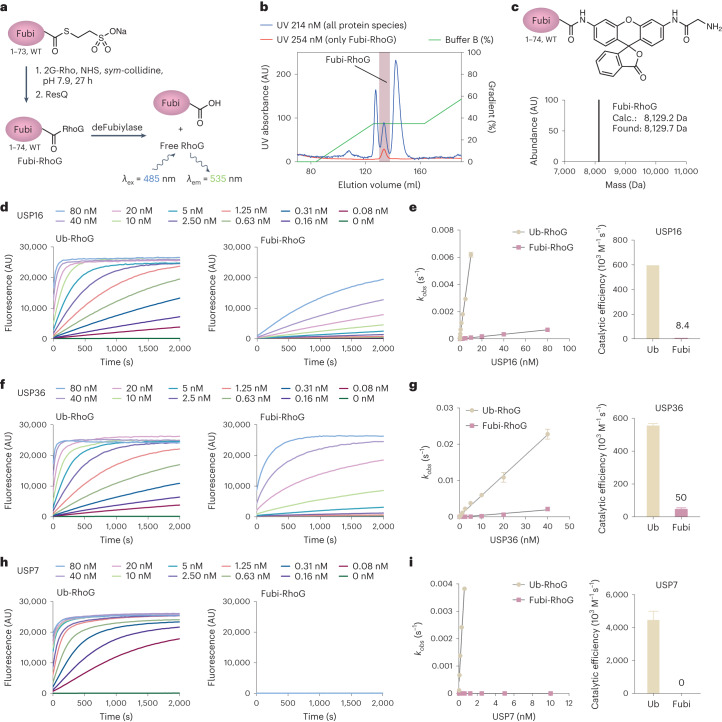
Quantification of ubiquitin and Fubi C-terminal hydrolase activity of USP DUBs with a fluorogenic Fubi-RhoG substrate. **a**, Schematic representation of the semisynthesis of the fluorogenic substrate Fubi-RhoG and its enzymatic turnover; 2G-Rho, 2G-Rhodamine; NHS, *N*-hydroxysuccinimide. **b**, Purification of Fubi-RhoG through optimized anion exchange chromatography. Fubi-RhoG-containing fractions are indicated in light purple and were evident from the 254-nm UV absorbance signal as Fubi lacks tryptophan and tyrosine residues; AU, arbitrary units. **c**, Structure and intact protein MS analysis of purified Fubi-RhoG; Calc., calculated. **d**–**i**, Quantification of ubiquitin/Fubi C-terminal hydrolase activity. Ubiquitin-RhoG or Fubi-RhoG (at 100 nM) were incubated with catalytic domains of the indicated purified enzymes, and fluorescence emission was recorded for USP16 (**d**), USP36 (**f**) and USP7 (**h**). Data are shown as the averages of three replicates. Observed rate constants were then plotted over enzyme concentrations to determine catalytic efficiencies for ubiquitin-RhoG and Fubi-RhoG processing by USP16 (**e**), USP36 (**g**) and USP7 (**i**). Data are shown as the results of curve fitting with associated s.e.m. Results were confirmed in three independent experiments. Source data

**Extended Data Fig. 3:**
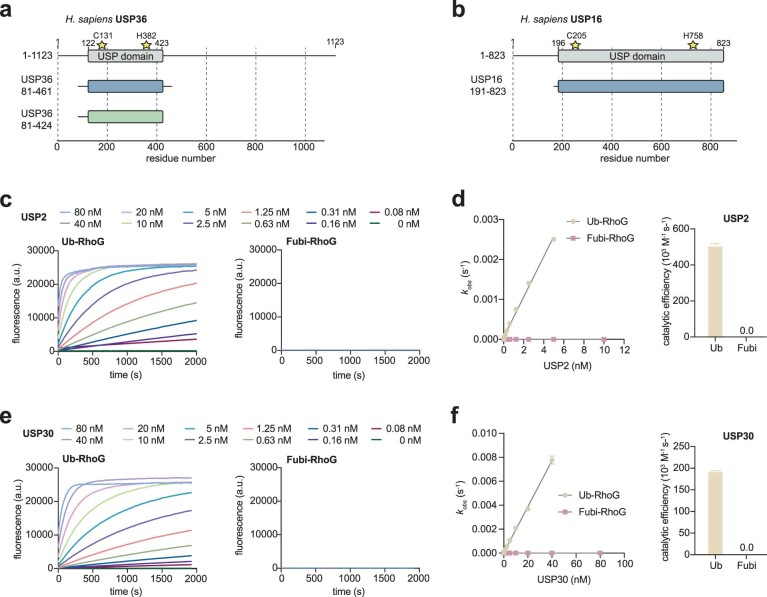
Quantification of Ubiquitin and Fubi C-terminal hydrolase activity of Ubiquitin-specific deubiquitinases. **a**. Schematic representation of human USP36 domain architecture with active site residues shown as stars. The protein boundaries used for crystallography studies are indicated. **b**. Schematic representation of human USP16 domain architecture. The protein boundaries used for in-vitro studies are indicated. The USP domain of USP16 contains various large insertions^50^. **c.-f**. Quantification of Ubiquitin and Fubi C-terminal hydrolase activity using Ub-RhoG and Fubi-RhoG substrates, respectively, for both USP2 (**c**, **d**) and USP30 (**e**, **f**) as described in Fig. 2d–i. Source data

To better understand this unusual ubiquitin/Ubl modifier dual activity of the identified USP DUBs, we next sought to quantify their ubiquitin and Fubi C-terminal hydrolase activities in vitro. Analogous to the widely used ubiquitin-rhodamine-monoglycine (Ub-RhoG) substrate^49^, we prepared a fluorogenic Fubi-RhoG substrate that liberates highly fluorescent RhoG after enzymatic turnover (Fig. 2a). Key to its purification in a native form (that is, without purification in acetonitrile-containing solvents and thus without the need for refolding) was a fine-tuned scouting of ion exchange chromatography conditions to separate it from other protein species formed during the reaction, such as free Fubi (Fig. 2b). In accordance with the considerably more hydrophobic nature of Fubi than ubiquitin (Supplementary Table 1), Fubi-RhoG was found to have limited solubility in aqueous buffers and could not be concentrated to more than 5 µM without partial aggregation; however, it was obtained as a pure and functional reagent (Fig. 2c–i). Kinetic analysis demonstrated enzyme concentration-dependent and complete turnover of Fubi-RhoG by recombinant USP36 (Extended Data Fig. 3a), yielding catalytic efficiencies for ubiquitin and Fubi (Fig. 2f,g). Recombinant USP16 (Extended Data Fig. 3b) processed the ubiquitin substrate with similar efficiency as USP36 and also cleaved Fubi-RhoG, albeit at a lower efficiency (Fig. 2d,e). Control DUBs USP7 (Fig. 2h,i), USP2 and USP30 (Extended Data Fig. 3c–f) only processed the ubiquitin substrate. These data demonstrate the dual ubiquitin/Fubi cleavage activity of USP16 and USP36.

**Fig. 3:**
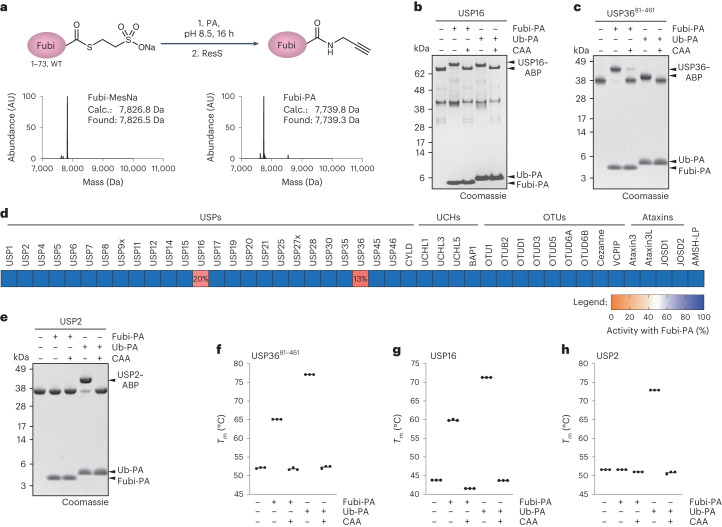
Assays of a panel of recombinant DUBs for Fubi-PA reactivity confirmed USP16 and USP36 as dual ubiquitin/Fubi-specific enzymes. **a**, Semisynthesis of the Fubi-PA probe from Fubi-MesNa (top) and characterization of these species by intact protein MS (bottom). **b**, Reactivity assessment of recombinant USP16 incubated with the indicated probes for 1 h at 37 °C. Chloroacetamide (CAA) pretreatment was performed where indicated. Protein samples were analyzed by SDS–PAGE and Coomassie staining; ABP, activity-based probe. **c**, Reactivity assessment of recombinant USP36 as described in **b**. **d**, Results of a DUB panel inhibition assay shown as a heat map. Recombinant DUBs were incubated with Fubi-PA (4 µM) for 15 min at room temperature, and their activities were subsequently assessed. Residual activities for USP16 and USP36 are given as numbers. Data show results from technical duplicates normalized for each DUB to its respective activity in the absence of probe; UCHs, ubiquitin carboxy-terminal hydrolases; OTUs, ovarian tumor family of DUBs. **e**, Reactivity assessment of recombinant USP2 as described in **b** and **c**. **f**–**h**, Thermal stability assessment of USP36 (**f**), USP16 (**g**) and USP2 (**h**) complexed with Fubi or ubiquitin probes. Means and individual results of three samples are plotted; *T*_m_, protein melting temperature. Source data

To further substantiate that the Fubi protease activity of these DUBs is unique among human DUBs, we screened a commercial panel of recombinant DUBs, which is normally used for screening of small-molecule inhibitors, for reactivity with a Fubi probe. For this, we prepared untagged Fubi-PA (Fig. 3a) and verified its reactivity with both recombinant USP16 and USP36 (Fig. 3b,c). Consistent with the chemoproteomics experiment (Fig. 1c), only USP16 and USP36 showed near-complete inhibition by Fubi-PA (Fig. 3d), whereas all other DUBs in the panel did not show a reduction in activity. Consistently, recombinant USP2 only reacted with the ubiquitin-based, but not the Fubi-based, probe (Fig. 3e). Binding of USP36 or USP16 to Fubi-PA led to pronounced protein stabilization as assessed by an increase in melting temperature, while binding to ubiquitin-PA for USP36, USP16 and USP2 correlated with even larger protein stability (Fig. 3f–h). These strong increases in protein melting temperature can be explained by recognition of Fubi by USP16 and USP36. The lower stabilization by Fubi than ubiquitin, indicative of weaker binding, correlated with the higher activity on the ubiquitin-based fluorogenic substrate.

**Extended Data Fig. 4:**
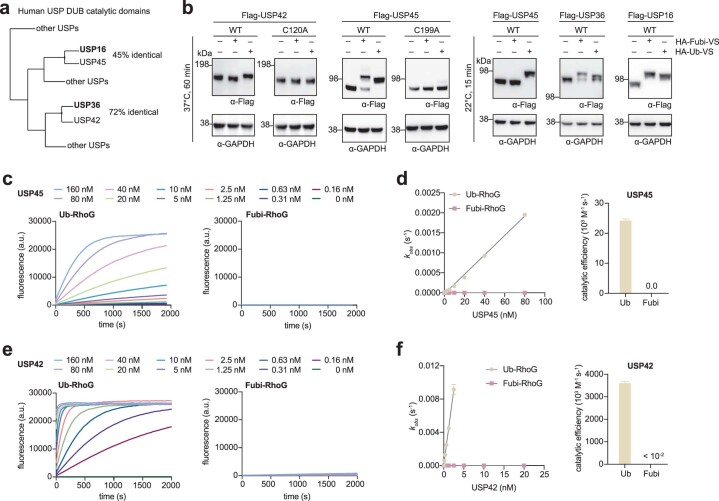
Assessment USP42 and USP45 as closest homologues of USP36 and USP16, respectively, in Fubi probe and Fubi-RhoG substrate assays. **a**. Focused homology tree of USP core catalytic domain sequences (that is, without insertions) according to Ye et al.^50^ Sequence identity and similarity between the two pairs of closely related DUBs are given. Compare Supplementary Fig. 1 in reference^51^. **b**. Assessment of USP42 and USP45 probe reactivity. Flag-tagged, full-length USP proteins were overexpressed in HEK293 cells and analyzed as in Fig. 1d and Extended Data Fig. 2c (*left*) or under milder conditions (*right*). Uncropped blots are provided as Source Data. **c.-f**. Quantification of Ubiquitin and Fubi C-terminal hydrolase activity using Ub-RhoG and Fubi-RhoG substrates, respectively, for both USP45 (**c**, **d**) and USP42 (**e**, **f**) as described in Fig. 2d–i and Extended Data Fig. 3c–f. Source data

We next investigated close homologs to both DUBs. The USP family of DUBs is composed of approximately 50 members^50^, which can be grouped into pairs or triplets according to highly related catalytic domain sequences^51^. USP16 and USP36 cluster together (Extended Data Fig. 4a); however, even more closely related homologs exist (USP42 shares 72% sequence identity with USP36, and USP45 shares 45% sequence identity with USP16). We assessed probe reactivity of full-length USP42 and USP45 in cell lysates and observed ubiquitin-specific probe binding for USP42 and partial reactivity of USP45 with HA–Fubi-VS (Extended Data Fig. 4b). However, under milder labeling conditions, USP45 showed ubiquitin-specific probe binding, while USP16 and USP36 retained dual Fubi- and ubiquitin-VS reactivity. Moreover, full-length recombinant USP45 did not show Fubi C-terminal hydrolase activity (Extended Data Fig. 4c,d), and insect cell-derived full-length USP45 was also not inhibited by Fubi-PA (Fig. 3d). USP42 showed barely detectable levels of Fubi-RhoG turnover (Extended Data Fig. 4e,f), demonstrating that despite being the closest homolog of USP36, USP42 does not react with Fubi in vitro or in cells.

### Structure and substrate recognition of USP36

**Fig. 4:**
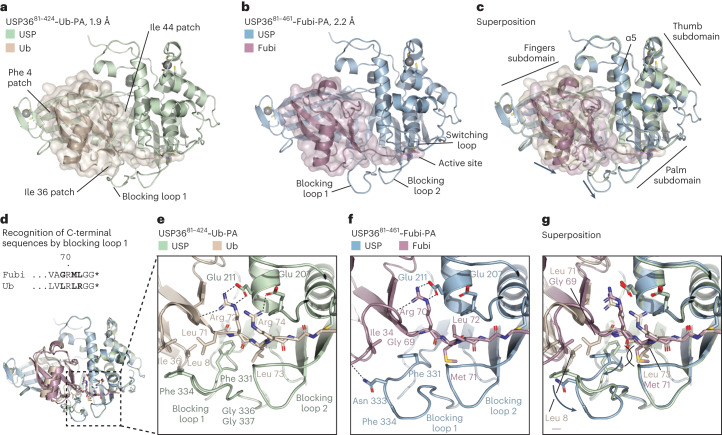
USP36 differentially interacts with the ubiquitin and Fubi C termini. **a**, Crystal structure of the USP36 catalytic domain (green) bound to the ubiquitin-PA probe (wheat). **b**, Crystal structure of the USP36 catalytic domain (blue) bound to the Fubi-PA probe (lilac). **c**, Superimposition of cartoon representations shown in **a** and **b**. USP subdomains and the α5 helix are labeled. Arrows indicate differences between Fubi- and ubiquitin-bound complexes. **d**, Depiction of Fubi and ubiquitin C-terminal sequences. **e**–**g**, Close-up view of the area shown in **d** where the C-terminal tail of ubiquitin (**e**) or Fubi (**f**) is guided to the catalytic center. A superposition is shown in **g**. Residues of USP36, Fubi and ubiquitin are labeled in colors according to the structure from which they are shown. Different orientations of residues in the USP36 blocking loop 1 after engagement of Fubi or ubiquitin are highlighted with black arrows.

**Extended Data Fig. 5:**
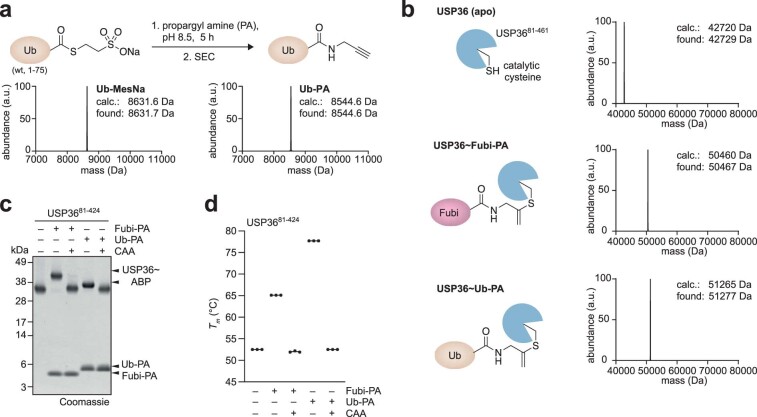
Preparation of covalent complexes of USP36 with Fubi and Ubiquitin probes for crystallography. **a**. Semi-synthesis of Ub-PA probe from Ub-MesNa (*upper part*) and characterization of these species by intact protein mass spectrometry (*lower part*). **b**. Intact mass spectrometry analysis of USP36 binding to Fubi- and Ub-PA probes, confirming 1:1 stoichiometry. **c**. Reactivity assessment of the shorter USP36^81–424^ construct with indicated probes as shown for the longer USP36 construct in Fig. 3c. The uncropped gel is provided as Source Data. **d**. Stability assessment of the shorter USP36^81–424^ construct complexed with Fubi or Ub probes as shown for the longer USP36 construct in Fig. 3f. Means and individual results of three samples are plotted. *T*_*m*_, protein melting temperature. Source data

**Extended Data Fig. 6:**
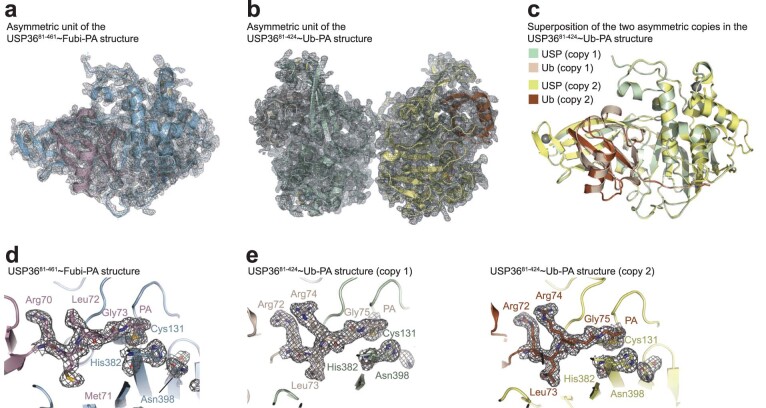
Structural characterization of specific Ubiquitin/Fubi cross-reactivity in USP36. **a**. Asymmetric unit of the USP36^81-461^~Fubi-PA structure. Protein chains are shown in cartoon representation with the electron density map overlayed in grey and contoured at 0.8 σ corresponding to the weighted |2F_0_-F_C_| electron density. **b**. Asymmetric unit of the of USP36^81-424^ ~ Ub-PA structure, shown as in **a**. **c**. Superimposition of the cartoon representations of two copies of the USP36~Ub-PA complex contained in the asymmetric unit of the USP36^81-424^ ~ Ub-PA structure. **d**. Zoom-in on the active site of USP36 in the USP36^81-461^~Fubi-PA structure showing the catalytic triad residues Cys131, His382 and Asn398 and the C-terminus of the Fubi-PA probe as sticks, overlayed with the electron density as in **a**. **e**. Zoom-in on the active sites of the two copies in the USP36^81-424^ ~ Ub-PA structure (density as in **b**, showing the C-terminus of the Ubiquitin-PA probe and the USP36 catalytic triad as in **d**).

We next asked which molecular determinants underlie Fubi cleavage activity in enzymes. While an NMR structure of free Fubi is available, how Fubi is specifically recognized is not known due to a lack of structures showing Fubi in complex with proteins. We focused on USP36 because a close homolog absent of Fubi activity exists, and its relatively compact catalytic domain seemed more amenable to structural analysis (Extended Data Fig. 3a,b). We purified USP36 in covalent complex with either Fubi-PA or ubiquitin-PA and obtained crystal structures at resolutions of 2.2 Å and 1.9 Å (Fig. 4a–c, Supplementary Table 2 and Extended Data Fig. 5a,b). The structure with ubiquitin-PA was solved using a USP36 construct lacking an unstructured sequence C terminal to the catalytic domain, which displayed unchanged reactivity and stability (Extended Data Fig. 5c,d). This structure featured two copies in the asymmetric unit that were completely superimposable, with excellent density covering all parts of the protein complexes (Extended Data Fig. 6a–e).

**Extended Data Fig. 7:**
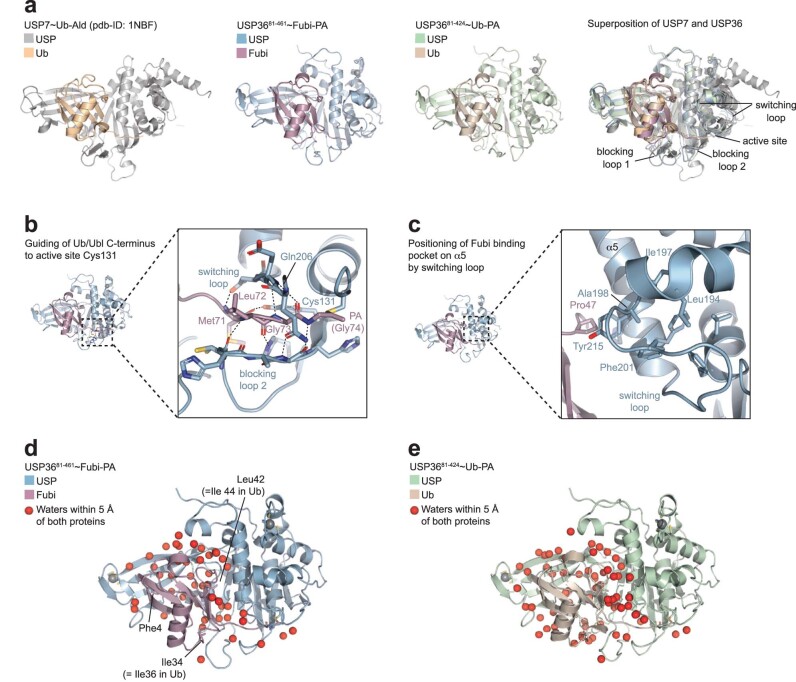
Substrate engagement by USP deubiquitinases. **a**. Cartoon representations of covalent USP-Ub/Ubl substrate complexes of USP7~Ubiquitin-aldehyde (*left*, PDB-ID: 1NBF), USP36~Fubi-PA (*second to left*), USP36~Ub-PA (*second to right*) and superposition of these three structures (*right*). Distinct conformations in the switching loops and in blocking loops 1 are highlighted. **b**. Zoom-in on the palm/thumb cleft of USP36 in the USP36~Fubi-PA structure, which guides the C-terminal tail of Fubi towards the active site Cys131. Hydrogen bonds are indicated with dotted lines. Gln206 of the switching loop contacts the blocking loop 2 backbone and thereby forms a narrow substrate channel, which is responsible for restricting cleavage activity to a terminal diGly motif. **c**. Zoom-in on the hydrophobic anchoring of the switching loop in USP36 on the α5 helix, stabilizing recognition of Fubi Pro47 by Tyr215 in USP36. **d**. Cartoon representation of the USP36~Fubi-PA structure with all waters which are within 5 Å of both proteins shown as red spheres. The large presence of water in the USP36-Fubi interface is typical for USP deubiquitinases^52^ and indicates numerous polar contacts. **e**. Cartoon representation as in **d** for the USP36~Ubiquitin-PA structure.

**Extended Data Fig. 8:**
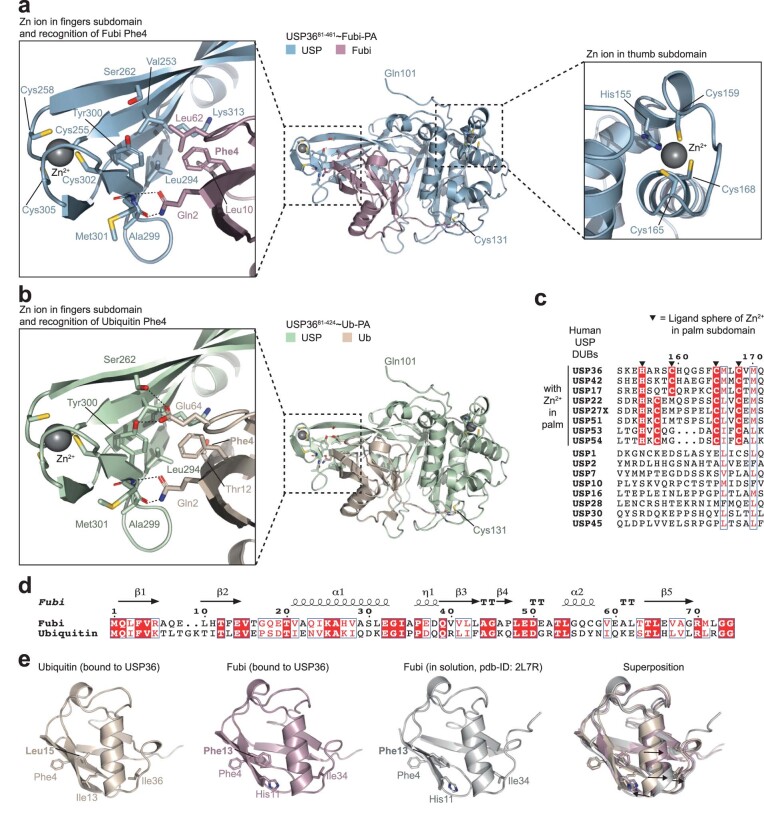
Substrate engagement by USP36. **a**. Cartoon representation of the USP36~Fubi-PA structure (*middle*) with zoom ins on the recognition of the Fubi Phe4 patch by the USP36 fingers subdomain (*left*) and on the additional zinc ion in the thumb subdomain (*right*). Residues shown as sticks are labeled. **b**. Cartoon representation of the USP36~Ubiquitin-PA structure showing the recognition of the Ubiquitin Phe4 patch by USP36, shown as in **a**. **c**. Sequence alignment of indicated human USP DUBs around the four residues of USP36 engaged in the coordination of the Zn ion in the thumb subdomain (colored with red background, residues shown in **a**) with sequences grouped according to the presence/absence of Zn coordinating motifs. **d**. Sequence alignment of human Fubi and Ubiquitin sequences with numbering and secondary structure assignments according to Fubi. Numbers of equivalent residues in Ubiquitin are shifted by 2 after the two amino acid deletion near the N-terminus of Fubi. **e**, Cartoon representations of Ubiquitin (*left*) and Fubi (*second to left*) from crystal structures, an NMR structure of free Fubi (*second to right*, PDB-ID: 2L7R) and superposition of these three structures (*right*). The distance between Phe4 and Ile36/Ile34 is larger in Fubi than in Ubiquitin due to the presence of bulkier Phe13 and His11 in the core of the Ubiquitin fold. The resulting shift of the helix and of the Ile34 patch is indicated with arrows to the right. An arrow at the bottom emphasizes the shorter loop resulting from the two amino acid deletion in Fubi within the Leu8 loop of Ubiquitin (compare **d**).

The catalytic domain of USP36 adopts the canonical USP fold^52^ comprised of thumb, palm and finger subdomains, which together as an S1 site recognize the β-sheet portion of the ubiquitin fold (Fig. 4c and Extended Data Fig. 7a–e). A narrow cleft between palm and thumb, flanked by two blocking loops and the switching loop, guides the C-terminal tail of the substrates into the active site through various hydrogen bonds (Extended Data Fig. 7a,b). The relative orientations of ubiquitin/Ubl modifier and USP as well as the USP fold itself are highly similar to other USPs, including USP7 (superposition with an r.m.s.d. of 0.9 Å over 327 Cα atoms), with four notable differences. (1) While blocking loop 1 in USP7 forms an antiparallel β-sheet commonly observed in USPs, the corresponding residues in both USP36 structures lack secondary structure. This allows for plasticity and a unique coordination of the C-terminal tail of Fubi, as described later (Fig. 4d–g). (2) The switching loop in USP36 is oriented toward the fingers and firmly engages the α5 helix through a hydrophobic cluster of Leu 194, Ile 197 and Phe 201 (Extended Data Fig. 7c). This enables Ala 198 to restrict the rotational freedom of the Tyr 215 side chain on α5, thereby forming a hydrophobic pocket toward the fingers subdomain, which is occupied by Pro 47 of Fubi. (3) While the backside of the USP domain in USP7 is engaged by a C-terminal extension, in USP36, an N-terminal extension starting at Gln 101 wraps around the domain, in line with our observation that N-terminal truncations lead to drastically reduced expression yields. (4) In addition to the commonly observed zinc ion coordination at the tip of the fingers, USP36 features a second zinc ion in the thumb subdomain made of CXXC and HX(XX)C motifs, which are present in eight human USPs (Extended Data Fig. 8a–c) and were previously only observed in structures of the DUB module in the yeast SAGA complex, which in humans comprises USP22 (refs. ^53,54^).

Recognition of ubiquitin is generally governed by three hydrophobic patches around Ile 44, Ile 36 and Phe 4 (ref. ^55^). Both ubiquitin and Fubi contact USP36 through a large, complementarily curved surface of around 1,700 Å^2^, which contacts all three patches (Fig. 4b,c) and also involves numerous water-mediated polar contacts (Extended Data Fig. 7d,e). Toward the fingers, both substrates are anchored on a hydrophobic surface spanned by Val 253, Leu 294, Tyr 300 and Lys 313 through their Phe 4 residues (Extended Data Fig. 8a,b). Distinct interfaces, however, exist around the other two patches.

The Ile 36 patch of ubiquitin, which also comprises Leu 71 and Leu 8, is recognized by USP36 in a canonical manner^52^ through Phe 334 as part of blocking loop 1 (Fig. 4d,e). The phenylalanine-in conformation of Phe 331 supports hydrophobic pockets for both Leu 71 and Leu 73, firmly anchoring the C-terminal tail, whereas Arg 72 and Arg 74 on the opposite side are contacted by glutamic acid residues. While Fubi does feature Ile 34 (equivalent to Ile 36 in ubiquitin), several sequence differences require a completely different recognition mode; substitution of Leu 15 and Ile 13 within the core of ubiquitin with larger Phe 13 and His 11 in Fubi shift the α-helix in both free and USP-bound Fubi (Extended Data Fig. 8d,e). This in turn moves the succeeding Ile 34-carrying loop of Fubi about 2.5 Å closer toward blocking loop 1 (Fig. 4c), which would be incompatible with Phe 334 in the ubiquitin-binding conformation (Fig. 4g). Instead, Phe 331 adopts a unique phenylalanine-out conformation in space that is generated through a Leu 71 to Gly 69 substitution in Fubi and through a two-amino-acid-long deletion around Leu 8. This conformational difference is facilitated by the glycine-rich blocking loop 1 sequence in USP36, which contains Asn 333, making polar contacts with the Fubi backbone, taking up the position of Phe 334. Moreover, Met 71 in Fubi replacing Leu 73 in ubiquitin is binding into a narrow hydrophobic pocket restricted by blocking loop 2 and thus likely helps to push Phe 331 into the out conformation (Fig. 4g). Last, Leu 72 in Fubi (replacing Arg 74 in ubiquitin) lacks the salt bridge to Glu 207 but mimics the hydrophobic interactions to neighboring residues (Fig. 4e–g). Overall, this comparison shows how conformational plasticity in blocking loop 1 of USP36 enables recognition of the distinctly different Ile 36/Ile 34 patches in ubiquitin and Fubi and thus assists in guiding of the scissile bond to the active site.

### Ubiquitin and Fubi cleavage specificity of USP DUBs

**Fig. 5:**
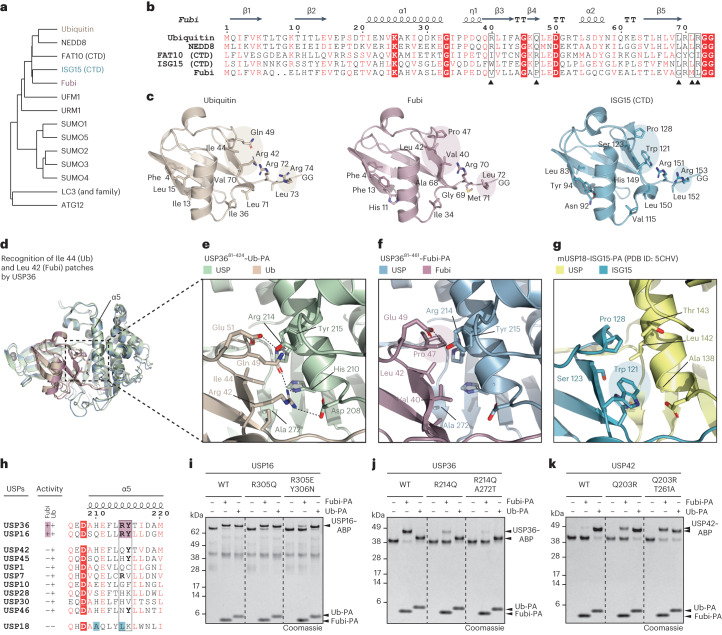
Molecular basis for Fubi/ubiquitin cross-reactivity. **a**, Average distance clustering of sequences from the shown human ubiquitin and Ubl modifier proteins; CTD, C-terminal Ubl domain. **b**, Sequences of ubiquitin and closely related Ubl modifiers. Numbering and secondary structure elements are shown according to Fubi. Numbers of equivalent residues in ubiquitin and other Ubl modifiers are shifted by two after the two-amino-acid-long deletion near the N terminus of Fubi. Black arrowheads indicate residues critical for Fubi recognition by USP DUBs, as validated by mutation. **c**, Cartoon representations of ubiquitin (left), Fubi (middle) and ISG15 (right) in their USP-engaged conformations. Various residues are shown as sticks and with the following labels: C-terminal tail residues (Leu 71, Arg 72, Leu 73 and Arg 74 for ubiquitin), hydrophobic patch residues (Phe 4, Ile 36 and Ile 44 together with Gln 49, Arg 42 and Val 70 for ubiquitin) and ubiquitin core residues (Ile 13 and Leu 15). Equivalent residues critical for ubiquitin/Ubl modifier recognition are shown in highlighted areas. **d**, Cartoon representation of USP36 in complex with ubiquitin and Fubi showing the area around the α5 helix that engages the Ile 44 (ubiquitin)/Leu 42 (Fubi) patches. **e**–**g**, Zoom-in images for the latter area for USP36 in complex with ubiquitin-PA (**e**), USP36 in complex with Fubi-PA (**f**) and mouse USP18 in complex with ISG15-PA^15^ (**g**); PDB, Protein Data Base. Hydrogen bonds are indicated as dotted lines. **h**, Sequence alignment of the α5 helix of the indicated human USP DUBs grouped according to ubiquitin/Ubl modifier cleavage activity as indicated by ‘+’ and ‘–’. The prospective Fubi recognition motif RY and Fubi activity are highlighted in purple. **i**, Probe reactivity assay for the indicated USP16 proteins analyzed by SDS–PAGE and Coomassie staining. **j**, Probe reactivity assay for the indicated USP36 proteins as in **i**. **k**, Probe reactivity assay for the indicated USP42 proteins as in **i**. Source data

Except for two aromatic residues (Phe 331 and Phe 334 in USP36), blocking loop 1 sequences are highly variable among USP DUBs^50^. We wondered what endows both USP16 and USP36 with the ability to recognize Fubi. We thus turned to the third hydrophobic patch around Ile 44 (Leu 42 in Fubi) and considered substrate specificity more broadly among Ubl modifiers (Fig. 5a,b). Cross-reactivity of USP DUBs for Fubi and ubiquitin is not obvious as Fubi has a higher similarity to ISG15 than ubiquitin, and for ISG15 the entirely specific USP member USP18 exists. While ISG15 shares the C-terminal LRLRGG with ubiquitin, its specific recognition by USP18 (ref. ^15^) relies on a different Ile 44 patch consisting of Ser 123, Pro 128 and Trp 121 instead of Ile 44, Gln 49 and Arg 42 in ubiquitin (Fig. 5c). USP36 engages Ile 44 in ubiquitin through His 210 and Arg 214, which is bent backward such that the aliphatic part of its side chain forms a hydrophobic surface (Fig. 5d,e). Gln 49 and Arg 42 engage Asp 208 of USP36 and the backbone of Leu 71 and thus assist in the positioning of ubiquitin through hydrogen bonding. By contrast, Fubi exhibits an entirely hydrophobic Leu 42 environment. Importantly, instead of polar Gln 49 in ubiquitin, Fubi is contacting USP36 primarily through Pro 47, which is tucked into a shallow hydrophobic pocket formed by two residues on the α5 helix, Arg 214 and Tyr 215, with the latter being oriented by the switching loop and by hydrogen bonding (Fig. 5f and Extended Data Fig. 7c). This mode of interaction is distinct from the recognition of ISG15 by USP18 (ref. ^15^), which contacts the equivalent Pro 128 with smaller side chains but instead engages larger Trp 121 (Val 40 in Fubi) through Ala 138 (His 210 in USP36; Fig. 5g and Supplementary Table 3).

Intriguingly, the presence of Pro 47-recognizing Arg 214 and Tyr 215 in USP36 correlates with Fubi reactivity, because also USP16, but neither the more closely related USP42, nor any other DUB tested, feature this motif (Fig. 5h). Consistently, mutation of the motif in USP16 reduced Fubi reactivity, while reactivity toward ubiquitin was retained (Fig. 5i). Disruption of the ‘RY’ motif also in USP36 lead to drastically reduced reactivity toward the Fubi probe (Fig. 5j). Excitingly, we found that introduction of an RY motif into USP42 generated reactivity toward Fubi, which is further increased by mutation of the only other residue different to USP36 on the probe binding surface (T261A; Fig. 5k). Collectively, our structural analysis shows how a sequence motif unique in Fubi-cleaving USP DUBs engages the more hydrophobic Ile 44 (Leu 42 in Fubi) patch environment of Pro 47 and Val 40 and also binds the more polar interface of ubiquitin.

**Extended Data Fig. 9:**
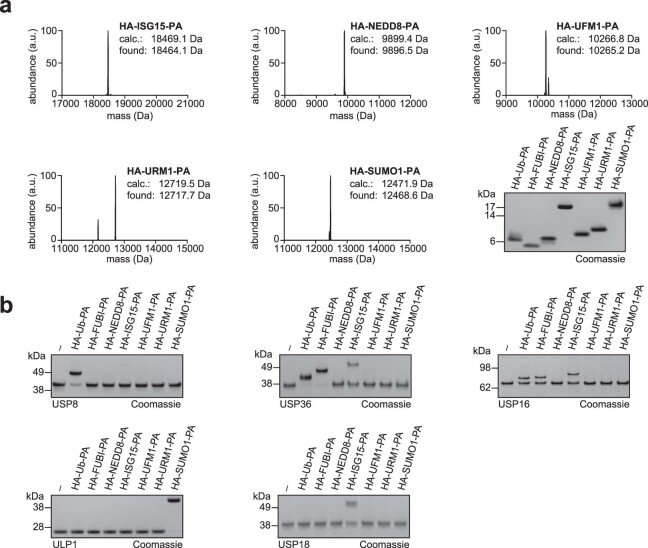
Comprehensive assessment of Ub/Ubl probe cross-reactivity in USP DUBs. **a**. Characterization of indicated HA-Ubl-PA probes by intact protein mass spectrometry and SDS PAGE. Uncropped gels are provided as Source Data. **b**. HA-Ub/Ubl-PA probe reactivity assay with indicated enzymes. Source data

Broader assessment of substrate specificity with a panel of Ubl modifier-PA probes (Extended Data Fig. 9a) revealed cross-reactivity of USP16 and USP36 with ISG15, which extends the number of ISG15-recognizing USP members (Extended Data Fig. 9b). This reactivity could be rationalized by equivalent prolines in Fubi and ISG15 (Fig. 5f,g). However, ISG15 reactivity does not automatically translate into Fubi reactivity as seen for USP2 and USP18 (Extended Data Fig. 9b). Recognition of ISG15 by Ubl modifier proteases appears to be based on a balance of various interactions^56^, and molecular determinants and potential roles of cross-reactive DUBs in interferon-related signaling will need to be worked out in future studies.

**Fig. 6:**
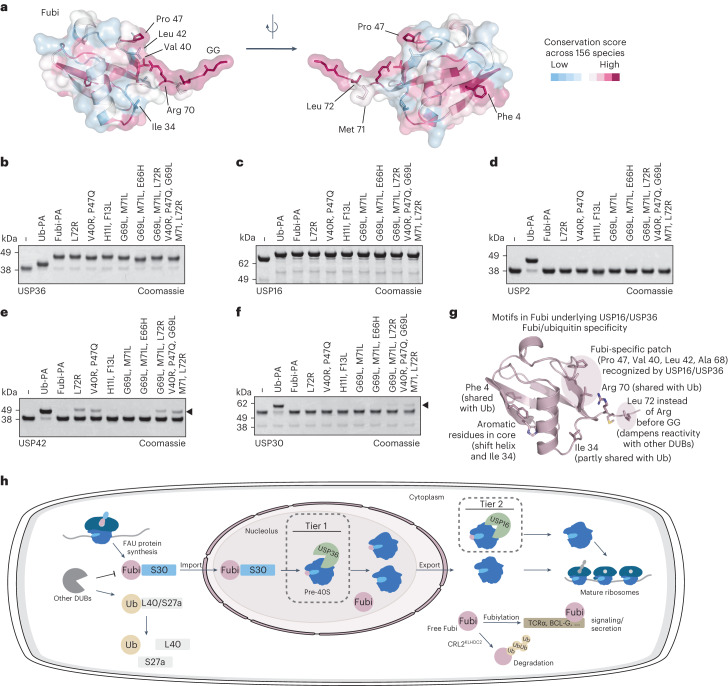
Validation of conserved sequence motifs in Fubi responsible for restricting cross-reactivity with USP DUBs. **a**, Cartoon and transparent surface representation of Fubi in two orientations colored according to sequence conservation, which was calculated from sequences of 156 species. Selected residues are shown as sticks and are labeled. **b**–**f**, Reactivity assessment of recombinant proteins USP36 (**b**), USP16 (**c**), USP2 (**d**), USP42 (**e**) and USP30 (**f**) with wild-type ubiquitin-PA, wild-type Fubi-PA or Fubi-PA probes carrying the indicated mutations (which substitute the ubiquitin-equivalent residues into Fubi). Cross-reactivity to mutated Fubi probes is shown with black arrowheads for USP42 and USP30. Data are representative of three independent experiments. **g**, Model summarizing important motifs on Fubi underlying the Fubi/ubiquitin cross-reactivity in USP16/USP36 and the restriction from other DUBs, thereby enabling spatially controlled maturation of Fubi-S30. **h**, Schematic representation of the proposed two-tier processing of Fubi-S30 and the Fubi system. Source data

**Extended Data Fig. 10:**
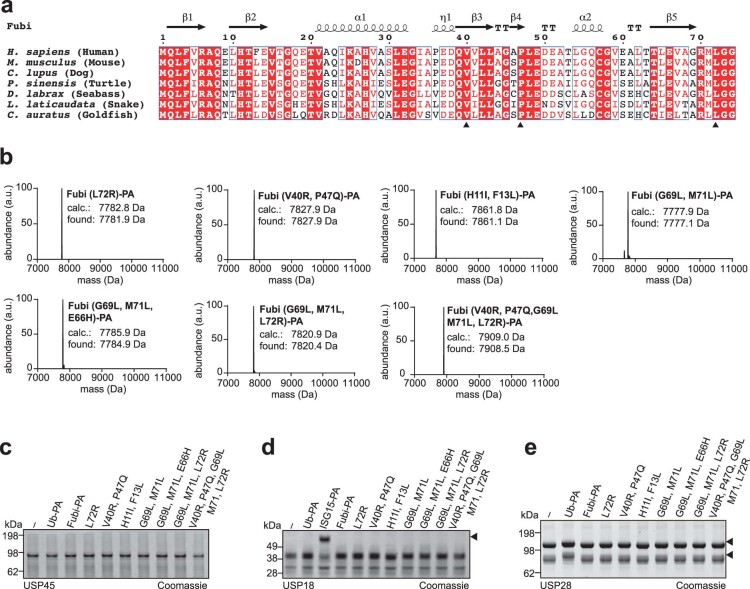
Assessment of molecular determinants of Fubi/Ub specificity. **a**. Sequence alignment of protein sequences of human Fubi and selected orthologues with secondary structure elements indicated according to the structure of free Fubi. **b**. Characterization of mutant Fubi-PA probes by intact protein mass spectrometry. **c.-e**. Ub/Ubl-PA probe reactivity assay. Wild-type Ub-PA, wild-type Fubi-PA, wild-type ISG15-PA (only for USP18) and mutant Fubi-PA probes were incubated with recombinant USP45 (**c**), USP18 (**d**) and USP28 (**e**). Uncropped gels are provided as Source Data. Source data

In contrast to ubiquitin, the sequence of Fubi has varied considerably during evolution (Extended Data Fig. 10a)^2,21^. Inspection of sequences from 156 different species selected as Fubi homologs due to their genomic location within the *S30* gene and filtered to cover 74 amino acids revealed strict conservation only for a few areas. These areas include the Phe 4 patch (required for binding to the fingers of USP36; Extended Data Fig. 8a), the C-terminal RXLGG tail unique among Ubl modifiers and strikingly both Pro 47 and Val 40 (Fig. 6a).

To experimentally test which of these areas restrict cross-reactivity of Fubi with other USPs, we assembled a set of mutated Fubi-PA probes where combinations of Fubi-specific residues were exchanged into their equivalent residues of ubiquitin (that is, with Fubi becoming more similar to ubiquitin; Extended Data Fig. 10b). In accordance with their dual ubiquitin/Fubi cleavage activity, USP16 and USP36 fully reacted with all probes (Fig. 6b,c), while USP2 reacted only with ubiquitin-PA but not with any of the Fubi-based probes (Fig. 6d). In line with its cleavage activity and homology to USP36, USP42 reacted with ubiquitin-PA and did not react with wild-type Fubi-PA but showed partial reactivity with several mutated Fubi-PA probes (Fig. 6e). These include probes with a single mutation of L72R (thus restoring the RXRGG ubiquitin C terminus) and a probe that restores the more polar Ile 44 environment (V40R and P47Q; Fig. 5f,k). More distantly related USP30 showed weak but consistently detectable reactivity with Fubi-PA probes mutated at both their C-terminal regions and toward a more Ubl Ile 44 environment (Fig. 6f), whereas USP18 retained its ISG15 specificity (Extended Data Fig. 10c–e).

These data demonstrate that cross-reactivity of Fubi toward other USP DUBs is firmly restricted by a Fubi-specific, entirely hydrophobic Ile 44 patch environment and a nonpolar residue preceding its C-terminal diglycine motif (Fig. 6g). This suppresses uncontrolled cleavage of Fubi-S30 by the bulk of the approximately 100 human DUBs and in turn enables its specific processing by USP16 and USP36.

## Discussion

Ribosomal biogenesis is a highly regulated process that involves transcription and processing of rRNA in the nucleolus, the stepwise incorporation of proteins assisted through assembly factors and the formation of translationally competent ribosomes in the cytoplasm^5,57^. Beyond the maturation of UBA52 and UBA82, the ubiquitination machinery aids in this process through clearance of excessive ribosomal proteins and through regulatory monoubiquitination^45,57^. USP36 is localized specifically to the nucleolus where it deubiquitinates various proteins essential for rRNA transcription and nucleolar structure^35–37^. Moreover, USP36 was recently reported to cleave the Fubi-S30 precursor and thus assist in the maturation of the small ribosomal subunit through its Fubi protease activity^22^. N-terminally cleaved S30 is observed in mature ribosomes^58^ and is required for translational activity, as seen from studies with noncleavable precursor^22^. Here, we report on a comprehensive assessment of Fubi C-terminal hydrolase activity through Fubi-derived activity-based probes and identified USP16 and USP36 as the only Fubi-reactive, cysteine-dependent hydrolases in HEK293 cell lysates (Fig. 1). Accumulation of uncleaved Fubi-S30 was seen for the co-depletion of USP16 and USP36 but not for co-depletion of the non-Fubi-reactive DUB USP7 and USP36 compared to USP36 depletion alone (Fig. 1 and Extended Data Fig. 2).

The available data converge into a model (Fig. 6h) where USP36 acts as the primary Fubi protease by cleaving Fubi-S30 in the nucleolus before or during an early stage of 40S assembly^22^. After completion of further maturation steps and export into the cytosol, USP16, as part of advanced 40S assembly intermediates^45^, then has the capacity to cleave yet unprocessed Fubi-S30, thereby acting as a backup, for example, during reduced levels or reduced activity of USP36 or nucleolar stress. This is in line with a role of USP16 proposed as part of a late-stage quality control step of 40S maturation^45^. Free Fubi can then be either conjugated to substrates for signaling and secretion or cleared by the proteasome through ubiquitination by the CRL2^KLHDC2^ E3 ligase as part of a C-degron pathway^59^.

With both USP16 and USP36 having roles in ribosomal biogenesis that involve their DUB activity, we cannot exclude that increases of Fubi-S30 seen following their double depletion (Fig. 1e–h and Extended Data Fig. 2e) are a result of perturbed ribosome formation. However, the observations that USP16 (1) can act downstream of USP36 in the biogenesis of the small ribosomal subunit, supported by distinct localizations^35^ and late-stage 40S maturation defects in USP16-depleted cells^45^, (2) is part of pre-40S ribosomal complexes^22,45^, (3) shows Fubi probe reactivity in lysates in a catalytic cysteine-dependent manner and (4) exhibits the necessary and specific Fubi cleavage activity in vitro collectively strongly suggest that Fubi-S30 is indeed cleaved by USP16. Taken together, the data are consistent with a two-tier system of Fubi-S30 cleavage during ribosomal biogenesis relying on Fubi cleavage activity of both DUBs in cells (Fig. 6h).

USP16, and in some cases explicitly its DUB activity, has been widely studied in the context of cell cycle regulation^44^, gene expression^46^ and the immune response^48^, yet its second catalytic activity had so far escaped attention. It will be important to assess whether the Fubi C-terminal hydrolase activity described here contributes to roles beyond ribosomal maturation. It appears unlikely that USP36 as a strictly nucleolar protein would be responsible for globally antagonizing Fubiylation. Owing to its cytoplasmic localization, we speculate that USP16 as a deFubiylase may survey the cytosol for aberrant Fubiylation. Moreover, Fubi-conjugate cleavage activity was observed in (at least) two distinct environments after lysate fractionation^23^, followed by further work toward the unbiased identification of Fubi proteases^60,61^. The introduction and successful application of Fubi-based probes reported herein will enable the search for further enzymes with deFubiylase activity in other cell lines and tissues. In light of the immunomodulatory roles of Fubi conjugates^27,32–34^, the reported roles of USP16 in immune cells provide a starting point for further studies. In analogy to DUBs^11^, its discovery will likely open the door to the enrichment and systematic investigation of Fubi conjugates.

Similarly, USP36 has been widely studied^22,36,37,47,48^, yet how its catalytic domain is capable of combining activity against ubiquitin and another Ubl modifier and why Fubi is seemingly not processed by the majority of DUBs were not clear. Cross-reactivity of some human DUBs against ubiquitin and a Ubl modifier (for example, ISG15 for USP5 or NEDD8 for UCHL3)^61^ has been reported but so far has not been explained mechanistically or been functionally rationalized. Our structures of the catalytic domain of USP36 in complex with both ubiquitin and Fubi revealed how its cross-reactivity is facilitated at the molecular level. Our data support a general mechanism for Fubi reactivity in the USP DUB fold. Key to this are (1) high plasticity in the blocking loop, required due to a sterically more demanding ‘Ile 36 patch’ in Fubi, and (2) an ‘arginine-tyrosine’ sequence motif on the α5 helix present in both Fubi-reactive DUBs USP16 and USP36 but not in close homologs, which facilitates the binding of a conserved and Fubi-specific ‘Ile 44 patch’ environment. Of note, both regions were also identified to facilitate the ISG15 specificity of USP18, yet in a way that abolishes ubiquitin binding completely^15^. By contrast, a viral OTU DUB achieves polyubiquitin/ISG15 cross-reactivity by contacting a partially overlapping region on the ubiquitin fold around Arg 42-ubiquitin/Trp 123-ISG15, equivalent to Val 40-Fubi, through an N-terminal domain extension that is unique to viral proteins^10^. This mode of ubiquitin/Ubl modifier activity through a rigid S1 site is also distinct from viral PLpro enzymes, which use an S2 site to modulate polyubiquitin/ISG15 cleavage activity^13,14^, and from bacterial CE clan proteases, which use an S1 site with two variable loops to achieve dual ubiquitin/SUMO activity^9^.

The different conformations in blocking loop 1 of USP36 are enabled by conformational flexibility of Phe 331, which is strongly conserved in USP domains. A rotation of the equivalent residue in USP7 was seen after small-molecule inhibitor binding^62^, yet the orientation observed in the Fubi-bound form is distinct from the apo conformation and ubiquitin- and inhibitor-bound conformations. The arginine-tyrosine motif allows for the recognition of the more hydrophobic Fubi and more polar ubiquitin environments. Protein stabilization (Fig. 3) and kinetic (Fig. 2) data suggest that Fubi is recognized by both USP16 and USP36 with lower affinity than ubiquitin. The rather poor engagement of Val 40 (Fig. 5) and lack of polar interactions of Leu 72 in Fubi instead of Arg 74 in ubiquitin would provide a rationale for this observation, yet these Fubi-specific changes also explain why Fubi is not recognized by even the most homologous DUBs, including USP42 (Fig. 6).

In the context of the ubiquitin/Fubi cross-reactivity of USP16, we find it intriguing that the Ovaa lab reported DUB-selective substrates^63^ for USP16, which were derived through point mutations on ubiquitin. In the most USP16-selective sequences, mutations are observed that resemble the characteristics of Fubi. These include the introduction of a large and polar residue into the core of ubiquitin (I13R, equivalent to the conserved His 11 in Fubi), a hydrophobic residue at Glu 64 (E64F, equivalent to Leu 62 in Fubi) and a remodeled C terminus (L71A, equivalent to Gly 69 in Fubi, and R74W and R74M, equivalent to Leu 72 in Fubi). It appears likely that this chemical evolution toward a DUB-selective substrate^63^ was possible as the substrate was becoming more Fubi-like, which prevented high activity by other DUBs through a mechanism also adopted by nature.

A comprehensive phylogenetic analysis concluded that the fusion of ubiquitin N terminal to the *S30* gene through exon shuffling and its subsequent divergence into a Ubl sequence occurred in three independent cases during evolution^21^. Fubi formed as one of these cases during metazoan evolution with characteristics (I13H, Q49P and loss of most lysine residues) recognized in its specific binding to USP36. It is not entirely clear why ubiquitin fusions N terminal to ribosomal proteins have evolved, but they act as chaperones and increase protein yield^22,57^. S30 has a particularly strong amino acid composition bias with 34% of basic (lysine and arginine) residues, and it is plausible that the oppositely charged Fubi protein compensates for this bias. In addition, the evolutionary divergence from ubiquitin of a Ubl modifier N terminal to S30 and the presence of specific Fubi protease activity in two DUBs implicated in ribosomal small subunit biogenesis imply a need for the coupling of Fubi-S30 ribosomal incorporation to its maturation and suggest that unregulated co- or post-translational processing, as is assumed for UBA52 and UBA82, would not be favorable for Fubi-S30 (Fig. 6h). Our mechanistic findings reveal how this is facilitated by a Ubl sequence that restricts activity from the majority of human DUBs and how a small subset of USP DUBs have been co-opted to provide specific and spatially defined ubiquitin/Fubi activity. Moreover, the chemical tool kit presented herein and the identification of a cytosolic deFubiylase can be expected to fuel the systematic investigation of Fubiylation as a post-translational modification.

## Methods

### Cloning, protein expression and purification

Human Fubi^1–73^, HA–Fubi^1–73^, GST–3C-GS-HA–Fubi^1–73^, ubiquitin^1–75^, HA–ubiquitin^1–75^, HA–NEDD8^1-75^, HA–ISG15^1–156^, HA–UFM1^1–82^, HA–URM1^1–100^ and HA–SUMO1^1–96^ were cloned from *Escherichia coli* optimized DNA gene strings into pTXB1 using restriction enzymes. cDNA encoding catalytic domains of human USP16^191–823^ (Medical Research Council Protein Phosphorylation and Ubiquitylation (MRC PPU), DU25374), USP42^70–446^ (MRC PPU, DU15140), USP36^81–461^ and USP36^81–424^ (MRC PPU, DU49030) were cloned into pOPINK, and USP2^258–605^ (Addgene, 22577), USP8^734–1110^ (Addgene, 22608) and USP18^16–372^ (MRC PPU, DU14320) were cloned into pOPINB using an In-Fusion HD cloning kit (Takara Clonetech). USP45^1–814^ was expressed from a pGEX6P vector (MRC PPU, DU15681). USP7^1–1102^, USP28^1–1077^ and USP30^64−Δ(box-2/3-insertion)-Δ(box-4/5-insertion)-502^ were expressed from previously published constructs^51,64,65^. For mammalian cell expression, full-length cDNA sequences for Flag–USP7, Flag–USP10 (from MRC PPU, DU15100), Flag–USP16, Flag–USP36, Flag–USP42 and Flag–USP48 (from MRC PPU, DU37160) were cloned into pOPINE. USP45 was expressed from pCMV-Flag-USP45 (MRC PPU, DU14306). Full-length Fubi-S30 was expressed with an N-terminal HA tag and a C-terminal Flag tag from pOPINE. Point mutations were generated using site-directed mutagenesis.

Bacterial expression was performed in Rosetta2(DE3)pLacI cells. Starter cultures were prepared the day before and diluted 1:100 in 2xTY medium supplemented with appropriate antibiotics. Cultures were grown at 37 °C with shaking at 180 r.p.m. until an optical density of 0.8–1.2 was reached, after which cultures were cooled to 18 °C, and expression was induced by adding isopropyl-1-thio-β-d-galactopyranoside (IPTG) to a final concentration of 0.5 mM. USP45 was expressed at 15 °C with 0.25 mM IPTG. After overnight growth, cells were collected by centrifugation and stored at −80 °C.

Ubiquitin, Fubi and other Ubl thioesters were obtained using intein-mediated C-terminal functionalization, chitin-binding affinity purification and size exclusion. Lysis was performed on ice by sonication in buffer A (20 mM HEPES, 50 mM sodium acetate and 75 mM NaCl, pH 6.5) supplemented with DNase. Protease inhibitors were included in the lysis buffer for Fubi and all other Ubl modifiers, and 5% glycerol was present in all buffers for Fubi and NEDD8 purification and functionalization. Lysate was cleared by centrifugation (22,500*g*, 30 min, 4 °C) and filtering (0.45-µm filter). Protein binding to the chitin column was conducted at room temperature for 30 min with gentle agitation for ubiquitin, Fubi and NEDD8 and at 4 °C with overnight binding for all other Ubl modifiers. Fubi was found to be sensitive to aeration; thus, gentle mixing was critical. Unbound proteins were washed away using a high-salt buffer (20 mM HEPES, 50 mM sodium acetate and 500 mM NaCl, pH 6.5). Proteins were eluted using 100 mM MesNa in buffer A for either 20 h at room temperature (ubiquitin) or 16–62 h at 4 °C (Fubi and other Ubl modifiers). For all GST–3C-GS-HA–Fubi proteins, the tag was removed by incubation (4–24 h) with 3C protease. The eluate was concentrated to 2.5 ml using Amicon Ultra-4 3,000 Da molecular weight cutoff concentrators and further purified in buffer A on a HiLoad 16/600 Superdex 75-pg column (GE Healthcare). For NEDD8, 0.05% (vol/vol) hydrazine monohydrate was added to the filtered eluate, and gel filtration was performed in 20 mM MES (pH 6.0).

USP2, USP8, USP16, USP18 and USP42 were purified as follows. All purification steps were performed on Äkta Pure systems (GE Healthcare). Cell pellets were lysed on ice using sonication in 50 mM H_2_NaPO_4_, 300 mM NaCl, 20 mM imidazole and 4 mM β-mercaptoethanol (pH 8.0) supplemented with lysozyme and DNase. After lysate clearing, as described above, proteins were purified on a 5-ml HisTrap column and eluted through a gradient into elution buffer (50 mM H_2_NaPO_4_, 300 mM NaCl, 500 mM imidazole and 4 mM β-mercaptoethanol, pH 8.0). Fractions were pooled, and the tag was removed via overnight incubation with 3C protease during dialysis into the appropriate buffers. USP16 and USP42 fractions were dialyzed into nickel binding buffer (50 mM H_2_NaPO_4_, 300 mM NaCl, 20 mM imidazole and 4 mM β-mercaptoethanol, pH 8.0) and further purified by reverse Ni-NTA chromatography. USP18 was dialyzed into anion exchange low-salt high-pH buffer (25 mM Tris (pH 9.0), 50 mM NaCl and 4 mM DTT) for purification by ion exchange, and USP2 was not dialyzed. The reverse nickel flow-through fractions of USP16 and USP42 were then diluted into anion exchange low-salt buffer (25 mM Tris (pH 8.5), 50 mM NaCl and 4 mM DTT) and cation exchange low-salt buffer (20 mM H_2_NaPO_4_ (pH 8.0), 50 mM NaCl and 4 mM DTT), respectively. USP16, USP18 and USP42 were further purified by ion exchange chromatography. The diluted USP16 and dialyzed USP18 protein samples were loaded onto a 6-ml Resource Q column, followed by gradient elution into anion exchange high-salt buffer (25 mM Tris (pH 8.5), 500 mM NaCl and 4 mM DTT for USP16) and anion exchange high-salt high-pH buffer (25 mM Tris (pH 9.0), 500 mM NaCl and 4 mM DTT for USP18). The diluted USP42 protein sample was loaded onto a 6-ml Resource S column, followed by elution into cation exchange high-salt buffer (20 mM H_2_NaPO_4_ (pH 8.0), 500 mM NaCl and 4 mM DTT). USP2, USP16 and USP18 were further purified on a HiLoad 16/600 Superdex 75-pg column using SEC buffer (20 mM Tris (pH 8.0), 100 mm NaCl and 4 mM DTT). USP8 was further purified on a HiLoad 16/600 Superdex 75-pg column in 20 mM HEPES (pH 7.0), 200 mm NaCl, 5 mM DTT and 5% (wt/vol) glycerol.

The cell pellet of USP45 was lysed on ice using sonication in 50 mM HEPES (pH 7.5), 300 mM NaCl and 1 mM TCEP supplemented with lysozyme and DNase. After lysate clearing, as described earlier, the protein was purified on a 5-ml GSTrap column and eluted through a gradient into buffer (50 mM HEPES (pH 8.0), 300 mM NaCl, 10 mM reduced glutathione and 1 mM TCEP). USP45 was further purified on a HiLoad 16/600 Superdex 200-pg column into SEC buffer.

USP36 was purified by Ni-NTA chromatography and cation exchange. After cell lysis and HisTrap purification, as detailed earlier for the other USP proteins (reverse Ni-NTA chromatography was not performed), the tag was removed by 3C protease during dialysis into 300 mM NaCl, 25 mM H_2_NaPO_4_ (pH 8.4), 4 mM DTT and 5% glycerol. Cation exchange was performed by diluting the USP36 solution with buffer (25 mM H_2_NaPO_4_ (pH 8.4), 4 mM DTT and 5% glycerol) to a final salt concentration of 60 mM NaCl immediately before loading onto the exchange column. To elute USP36, a high-salt buffer (25 mM H_2_NaPO_4_ (pH 8.4), 500 mM NaCl, 4 mM DTT and 5% glycerol) was applied as a gradient.

USP7, USP28 and USP30 were purified as described previously^51,64,65^. Yeast ULP1 (residues 403–621 in pET19; UniProt: Q02724) was obtained from the Dortmund Protein Facility. Protein concentrations were determined using a NanoDrop or Bradford assay, and purity was assessed by intact LC–MS and SDS–PAGE. Purified proteins were snap-frozen in liquid nitrogen and stored at −80 °C.

For the crystallography sample of USP36^81-461^~Fubi-PA, USP36^81–461^ was purified by HisTrap affinity chromatography, followed by reverse HisTrap chromatography, and the complex was formed by adding Fubi-PA as titrated in preexperiments for complete binding and incubation for 1 h at room temperature. The reaction was then dialyzed into 25 mM H_2_NaPO_4_ (pH 8.4), 50 mM NaCl, 4 mM DTT and 5% glycerol, purified by cation exchange chromatography, as detailed earlier, and by size-exclusion chromatography and concentrated in 20 mM Tris (pH 8.0), 100 mM NaCl and 5 mM DTT. USP36^81-424^~ubiquitin-PA was obtained as described above from USP36^81–424^ and ubiquitin-PA.

### Crystallography

Crystallization experiments were set up in 96-well sitting drop vapor diffusion plates (MRC format, Molecular Dimensions) at 20 °C. Plates for fine screens were prepared using a Dragonfly robot (TTP Labtech). Drops were generated using the Mosquito HTS robot (TPP Labtech). For coarse screens, drops were created with 150 nl of reservoir from commercially available plates and 150 nl of protein solution, and for fine screens, drops consisted of 600–800 nl using a 1:1 or 2:1 protein:buffer volume ratio. USP36^81-461^~Fubi-PA (8.5 mg ml^–1^) was crystallized in 0.1 M MMT buffer (pH 7.0; consisting of malic acid, MES and Tris in a 1:2:2 molar ratio) and 25% (wt/vol) PEG 1500. The crystal was cryoprotected in reservoir mixed with ethylene glycol (3:1 volume ratio). USP36^81–424^~ubiquitin-PA (9.2 mg ml^–1^) was crystallized in 0.3 M potassium formate and 14% (wt/vol) PEG 3350. The crystal was cryoprotected in reservoir mixed with glycerol (3:1 volume ratio). Crystal diffraction datasets were obtained at the Swiss Light Source (Paul Scherrer Institute) on the PX2 beamline. Images were integrated using Xia2/DIALS^66^ or XDS^67^, and scaling was performed with Aimless^68^. Structures were solved through molecular replacement with Phaser^69^ using an alphafold^70^ structure for USP36 (UniProt: Q9P275) and structures of ubiquitin (PDB: 1UBQ) and Fubi (PDB: 2L7R) as search models. The final models were obtained from several rounds of manual building in Coot^71^ as part of CCP4 (ref. ^72^) and refinement with Phenix.Refine^73^. See Supplementary Table 2 for final structure statistics. Protein structures have been deposited with the PDB and are listed as PDB IDs 8BS3 (USP36^81-461^~Fubi-PA) and 8BS9 (USP36^81-424^ ~ubiquitin-PA). Protein figures were generated with PyMOL.

### Preparation of activity-based probes

Fubi-, ubiquitin- and Ubl-MesNa thioesters were concentrated in buffer A (see below for NEDD8 probe generation). To generate probes with a PA warhead, the protein was combined with PA hydrochloride dissolved in buffer A (titrated with NaOH such that the final pH was 8.5; typical final concentrations for ubiquitin: 0.9 mM protein and 1.85 M PA; typical final concentrations for Fubi and other Ubl modifiers: 0.65 mM protein and 1.2 M PA) and incubated at room temperature. The reaction was monitored via intact protein LC–MS, continued until all thioester species were eliminated and typically completed within 5 h to 16 h. To generate probes with a VS warhead, a solution of VS-HCl (see Supplementary Methods) in buffer A was first combined with a solution of *N*-hydroxysuccinimide (in buffer A with pH adjusted to 8.4) in a 1:1 volumetric ratio with final concentrations of 200 mM each. Protein thioesters (200 µM) were added in a 1:1 volumetric ratio, and the reaction was monitored as described above. A mutation at Cys 57 in Fubi (C57L) was needed to suppress side reactions (for example, the addition of a Cys 57 thiol to a free VS molecule or the addition of a Cys 57 thiol to a warhead installed on another Fubi-VS protein). Ubiquitin-PA probes were purified by size exclusion on a HiLoad 16/600 Superdex 75-pg column (GE Healthcare) in buffer A. Anion exchange on a ResQ column was used for purification of the Fubi-VS and Fubi-PA probes in 20 mM HEPES, 50 mM NaCl and 5% glycerol (pH 7.0). For purification of the ubiquitin-VS probe, cation exchange on a ResS column was performed using 50 mM sodium acetate and 100 mM NaCl (pH 4.5), with gradient elution for both probes into buffer containing 500 mM NaCl. For the generation of HA–NEDD8-PA, HA–NEDD8-hydrazide (55 µM in 20 mM MES, 30 mM Tris (pH 7.5) and 100 mM NaCl) was cooled for 2 min in a salt-ice-water bath (–10 °C) before cold 1 M NaNO_2_ and 200 mM citric acid solutions were added in a 2:1:1 volumetric ratio and incubated for 5 min. Then, 500 mM PA hydrochloride in 1.5 M HEPES (pH 8.0) was added to the cold solution, which was directly warmed to 30 °C and incubated for 5 min. HA–NEDD8-PA was purified on a ResS column in 50 mM sodium acetate (pH 4.5) with an elution gradient ranging from 0 to 500 mM NaCl.

### DUB panel screen

The selectivity of the Fubi-PA probe across a panel of recombinant purified DUBs was analyzed through testing in the DUBprofiler panel at Ubiquigent using 4 µM Fubi-PA and a 15-min incubation at room temperature. Ubiquitin-Rhodamine substrate was then added, and fluorescence was recorded after a 40-min incubation at room temperature. Catalytic activity of all DUBs was apparent from increased fluorescence in all control samples. Testing was performed in technical duplicates, and the activity of each DUB was normalized to that of untreated enzyme, as per the standard protocol. Data were visualized in Microsoft Excel.

### Preparation of Fubi-RhoG

Fubi-MesNa (420 µM) in buffer A was reacted with 2G-Rhodamine (15 equiv. in DMSO), *N*-hydroxysuccinimide (15 equiv. in buffer A and the pH adjusted to 7.1) and *sym*-collidine (15 equiv.), added in this order, at pH 7.9 (adjusted with NaOH solution) for 27 h. The reaction was monitored by LC–MS as described above. Excess 2G-Rhodamine was removed by two rounds of dialysis of 16 and 3 h in 20 mM Tris (pH 7.5), 20 mM NaCl, 1 mM TCEP and 5% glycerol. Fubi-RhoG was then purified using anion exchange chromatography on a ResQ column in 20 mM Tris (pH 7.5), a gradient of 20 mM NaCl to 300 mM NaCl, 1 mM TCEP and 5% glycerol. The UV 254-nM signal uniquely emitted from the protein-coupled RhoG facilitated detection and accurate separation of Fubi-RhoG.

### Ubiquitin/Fubi-RhoG cleavage assays

Kinetic assays were performed in black, low-volume, non-binding surface 384-well plates by mixing 10 µl of 2× substrate (final concentration of 100 nM) and 10 µl of 2× enzyme (final concentration, as indicated, of 160–0.08 nM). Substrate turnover was monitored in a Tecan Spark plate reader by recording fluorescence (excitation of 485 nm and emission of 535 nm) every 30 s over 30–60 min at 30 °C. All assays were performed in the same buffer (20 mM Tris (pH 8.0), 0.01% Triton X-100, 0.1 mg ml^–1^ bovine serum albumin and 1 mM TCEP). Concentrations of ubiquitin-RhoG and Fubi-RhoG stocks were measured by a bicinchoninic acid (BCA) assay, and stocks were titrated to reach the same final fluorescence after complete conversion by USP36. Observed rate constants were determined through curve fitting (one-phase association) in GraphPad Prism.

### Thermal shift assay

USP proteins (at 4 µM in PBS and 4 mM DTT) were reacted with Fubi-PA or ubiquitin-PA (4 µM in PBS and 4 mM DTT) for 20 min at room temperature. Enzymes were pretreated with 20 mM CAA for 10 min at room temperature where indicated. The protein solution was then combined with SYPRO Orange dye (6× in PBS and 4 mM DTT) at a 1:1 volumetric ratio. Thermal denaturation was recorded in triplicate in the FRET channel as described previously^74^. Melting temperatures are given as the inflection point of the fluorescence over temperature curve.

### Recombinant protein probe labeling assay

Purified proteins were diluted in 20 mM Tris (pH 8.0), 300 mM NaCl, 2 mM DTT and 5% glycerol. The protease and probe were combined at 2.5 µM and 10 µM final concentrations, respectively, and reacted for either 15 min at room temperature (Fig. 5i,j) or 1 h at 37 °C (all other assays). Binding was then assessed by either intact LC–MS or SDS–PAGE. Ubiquitin/Ubl modifier probe panel assays (Extended Data Fig. 9) and the USP16 mutant assay (Fig. 5i) were performed with 1.25 µM protease and 5 µM probe, which were reacted for 15 min at room temperature.

### Intact protein mass spectrometry

Fubi-RhoG was diluted in a 1:1 mixture of buffer A and acetonitrile and filtered using Proteus Mini Clarification spin columns; all other proteins were injected directly as aqueous buffered solutions. Samples (300 ng to 1.5 µg) were applied to an AdvanceBio Desalting-RP column (0.4 ml min^–1^; solvent A: HPLC-grade water and 0.1% trifluoroacetic acid; solvent B: HPLC-grade acetonitrile and 0.1% trifluoroacetic acid; gradient of 5–80% B over 2.5 min with a column temperature of 32 °C). The analysis was performed on an Agilent 1260 II Infinity system equipped with an electrospray ion source and an MSD mass spectrometer recording spectra in positive mode (capillary voltage of 4 kV, desolvation gas flow of 11 liters min^–1^ and temperature of 350 °C). Mass spectra were deconvoluted using ProMass (Enovatia).

### Cell culture

HeLa, HEK293 and MCF7 cells were obtained from the Leibniz Institute DSMZ German Collection of Microorganisms and Cell Cultures and cultured in DMEM (high glucose, GlutaMAX). Media for all cells were supplemented with 10% fetal bovine serum and penicillin/streptomycin, and in MCF7 cells, the medium was also supplemented with 10 µg ml^–1^ human insulin. Cells were grown at 37 °C in a humidified atmosphere with 5% CO_2_. Cell counts and viability checks were performed using a Countess II (Invitrogen) and trypan blue staining. Cells were tested to be free of *Mycoplasma* contamination.

### Transfections

Cells were seeded at 7 × 10^5^ cells per well in six-well dishes. DNA transfections were performed using PEI transfection reagent (Polysciences). DNA (2 µg) and PEI (6 µg) were diluted in Opti-MEM (200 µl), and, after a 15-min incubation, the mixture was added dropwise to cells. siRNA transfections were performed using Lipofectamine RNAiMAX (Invitrogen). siRNA (2 µl of a 10 µM stock) was diluted in 98 µl of Opti-MEM and added to RNAiMAX (6 µl diluted in 94 µl of Opti-MEM). When depletion of two targets was required, 2 µl of each siRNA and 12 µl of RNAiMAX were used. Single-target depletions in these experiments were performed with supplementation with 2 µl of siControl. After an incubation time of 5 min, the mixture was added dropwise, and cells were collected after 48 h. The following Dharmacon siRNA pools were used: siControl (D-001206-14-05), siUSP7 (M-006097-01-0005), siUSP16 (M-006067-01-0005) and siUSP36 (M-006084-02-0005). Cells were typically collected with 50 mM Tris (pH 8.0), 300 mM NaCl, 1% IGEPAL, 5% glycerol, 2 mM DTT, cOmplete EDTA-free protease inhibitor cocktail, 1 mM phenylmethylsulfonyl fluoride, 2.5 mM EDTA, 2.5 mM NEM and benzonase for 15 min on ice. Lysed cells were centrifuged at 14,000*g* for 10 min at 4 °C and subsequently adjusted to a protein concentration of 3–4 mg ml^–1^. Proteins were then separated by SDS–PAGE.

### Activity-based protein profiling in lysates

Cells were washed with cold PBS and lysed directly with 50 mM Tris (pH 8.0), 150 mM NaCl, 1% IGEPAL, 5% glycerol and 2 mM DTT for 15 min on ice. For samples where USP36 detection was desired, the same buffer supplemented with an additional 150 mM NaCl and benzonase was used. Lysate at 2 mg ml^–1^ was then incubated with the indicated probe at 10 µM for 1 h at 37 °C. The reaction was quenched by the addition of LDS loading buffer (supplemented with 50 mM DTT) and analyzed by SDS–PAGE and western blotting.

### Western blotting

Samples were transferred to nitrocellulose (PVDF for Fubi blots) membranes using a Trans-Blot Turbo system (Bio-Rad). Membranes were blocked with 5% (wt/vol) nonfat milk in PBS-T (PBS and 0.1% Tween 20) and incubated overnight with the indicated primary antibodies (anti-FAU, 1:1,000, Proteintech, 13581-1-AP; anti-Flag M2, 1:2,000, Sigma, F3165; anti-HA, 1:1,000, BioLegend, 16B12; anti-GAPDH, 1:10,000, Thermo Fisher, AM4300; USP7, 1:2,000, Abcam, ab190183; USP16, 1:1,000, Biomol, A301-614A-T; USP36, 1:500, Biomol, A300-940A-T). The signal was developed with secondary antibodies coupled to horseradish peroxidase (anti-mouse, 1:5,000, Sigma, NXA931; anti-rabbit, 1:5,000, Sigma, GENA934) and Clarity Western ECL substrate (Bio-Rad) supplemented with Clarity Max Western ECL substrate (Bio-Rad) when necessary. Chemiluminescence was imaged on a Chemi-Doc MP Imaging System (Bio-Rad), and quantification was performed using Image Lab (Bio-Rad).

### Identification of probe-labeled proteins by mass spectrometry

HeLa cells were cultured to 90% confluency in a 15-cm dish, collected by scraping in PBS and snap-frozen as pellets in liquid nitrogen. Cells were lysed in 50 mM Tris (pH 8.0), 50 mM NaCl, 0.2% Triton X-100, 1.25 mM DTT and 0.5 mM phenylmethylsulphonyl fluoride for 20 min on ice. The sample was then sonicated for 10 s (1 s on and 1 s off) at 10% amplitude and centrifuged for 10 min at 14,000*g* and 4 °C. The supernatant (1 mg of total protein at 4.7 mg ml^–1^) was incubated with 5 µg of the HA-tagged probe for 1 h at 37 °C. Where appropriate, the lysate was pretreated with IAA for 15 min on ice at a final concentration of 10 mM. After labeling, the reactions were quenched through the addition of SDS (0.2% final concentration) and incubated with CAA (5 mM) for 20 min. The lysate was then diluted fourfold in immunoprecipitation buffer (50 mM Tris (pH 8.0), 50 mM NaCl and 0.2% Triton X-100) supplemented with cOmplete EDTA-free protease inhibitor cocktail, and immunoprecipitation was performed using EZview Red anti-HA affinity gel (25 µl of slurry per 1 mg of lysate). Beads were washed three times with immunoprecipitation buffer, washed three times with PBS and sequentially treated with 1 mM DTT and 5 mM CAA. The proteins were then digested with Lys-C and trypsin. Peptides were desalted with C18 StageTips and analyzed by nano-HPLC–MS/MS, as described previously^74^. Relative quantification of proteins was performed using MaxQuant^75^ (v.2.0.3.1), including the Andromeda search algorithm and searching in parallel the *Homo sapiens* reference proteome of the UniProt database and the sequences of HA–Fubi, HA–UFM1 and HA–ubiquitin. Briefly, an MS/MS ion search was performed for enzymatic trypsin cleavage, allowing two missed cleavages. Carbamidomethylation was set as a fixed protein modification, and oxidation of methionine and acetylation of the N terminus were set as variable modifications. The mass accuracy was set to 20 ppm for the first search and to 4.5 ppm for the second search. The false discovery rates for peptide and protein identification were set to 0.01. Only proteins for which at least two peptides were quantified were chosen for further validation. Relative quantification of proteins was performed by using the label-free quantification algorithm. Statistical data analysis of pulldown samples was performed using Perseus^76^ (v.1.6.14.0), for which label-free quantification intensities were log_2_ transformed, and replicate samples were grouped together. Missing values were imputed using small normally distributed values, and a two-sided *t*-test was performed (*s*_0_ = 1 and false discovery rate *P* value = 0.01).

### Homology alignments

Alignments were analyzed in Jalview and visualized with ESPript 3. Homology trees were calculated using the Neighbor Joining and BLOSUM62 matrix method. Sequence conservation was analyzed using the Consurf webserver^77^.

### Statistics and reproducibility

Statistical analysis in Fig. 1c was performed using a two-sided *t*-test as part of Perseus. All other graphs and statistical analyses were generated and performed using GraphPad Prism. Statistical significance for data shown in Fig. 1f,g was determined by individual one-sample, two-tailed *t*-tests (for Fig. 1f, comparison of USP36 to siScr: exact *P* = 0.0336, comparison of siUSP36 to siUSP36 + siUSP16: exact *P* = 0.0013; for Fig. 1h: comparison of USP36 to siScr: exact *P* = 0.0336, comparison of siUSP36 to siUSP36 + siUSP16: exact *P* = 0.0013). All observations were made in at least two (typically three to six) independent experiments. Specifically, probe labeling experiments in lysates were repeated twice (Fig. 4d, right, and Extended Data Fig. 2b,c) or three times (Figs. 1d and 4d, left, and Extended Data Fig. 2a). Fubi processing experiments in HEK293 cells were performed four times (Fig. 1e,g), and the equivalent experiment in MCF7 cells (Extended Data Fig. 2e) was repeated three times. For in vitro probe labeling experiments, three (Figs. 3b,c,e and 6b–f and Extended Data Fig. 10c–e) or two (Fig. 5i–k and Extended Data Figs. 5c and 9a,b) independent experiments were performed. Kinetic experiments (Fig. 2d–i and Extended Data Figs. 3c–f and 4c–f) were performed with three independent wells per condition per plate, and two independent repeats were performed for all data shown, which yielded consistent results. A representative set of data is shown. Similarly, thermal shift analysis was performed with three independent wells per plate per sample, and the experiment was repeated once more with identical results.

### Reporting summary

Further information on research design is available in the Nature Portfolio Reporting Summary linked to this article.

## Online content

Any methods, additional references, Nature Portfolio reporting summaries, source data, extended data, supplementary information, acknowledgements, peer review information; details of author contributions and competing interests; and statements of data and code availability are available at 10.1038/s41589-023-01388-1.

### Supplementary information

Supplementary Information Supplementary Tables 1–3, Note (Chemical Synthesis) and References.

Reporting Summary

### Source data

Source Data Fig. 1 Unprocessed western blots and/or gels.

Source Data Fig. 3 Unprocessed western blots and/or gels.

Source Data Fig. 5 Unprocessed western blots and/or gels.

Source Data Fig. 6 Unprocessed western blots and/or gels.

Source Data Extended Data Fig. 2 Unprocessed western blots and/or gels.

Source Data Extended Data Fig. 4 Unprocessed western blots and/or gels.

Source Data Extended Data Fig. 5 Unprocessed western blots and/or gels.

Source Data Extended Data Fig. 9 Unprocessed western blots and/or gels.

Source Data Extended Data Fig. 10 Unprocessed western blots and/or gels.

Source Data Fig. 1 Numerical source data.

Source Data Fig. 2 Numerical source data.

Source Data Fig. 3 Numerical source data.

Source Data Extended Data Fig. 1 Numerical source data.

Source Data Extended Data Fig. 3 Numerical source data.

Source Data Extended Data Fig. 4 Numerical source data.

Source Data Extended Data Fig. 5 Numerical source data.

Source Data Extended Data Fig. 9 Numerical source data

Source Data Extended Data Fig. 10 Numerical source data.

## Data Availability

Data have been deposited with the PDB (accession codes 8BS3 and 8BS9) and the ProteomeXchange (accession code PXD038455). Compound characterization data are provided in the Supplementary Information. This study used the following protein structures previously deposited with the PDB: accession codes 1UBQ, 2L7R and 1NBF. Protein sequences for yeast ULP1 and human USP36 are available through UniProt accession codes Q02724 and Q9P275, respectively. Source data are provided with this paper.
