# Supplementary material for: Molecular basis for ubiquitin/Fubi cross-reactivity in USP16 and USP36
Source: Nat Chem Biol. 2023 Jul 13;19(11):1394–405. doi: 10.1038/s41589-023-01388-1 (PMC10611586; doi:10.1038/s41589-023-01388-1)
Supplement: Supplementary file 1 — Supplementary Tables 1–3, Note (Chemical Synthesis) and References. [file 41589_2023_1388_MOESM1_ESM.pdf]

nature chemical biology

Article https://doi.org/10.1038/s41589-023-01388-1

Molecular basis for ubiquitin/Fubi cross-reactivity in USP16 and USP36

In the format provided by the authors and unedited

Table of Contents:

Supplementary Tables .... p. 3
Supplementary Note (Chemical synthesis) .... p. 6
Supplementary References .... p. 8

Supplementary Tables

Supplementary Table 1. Amino acid composition of Fubi and Ubiquitin.

	**Fubi**	**Ubiquitin**
Total # of residues	74	76
# positively charged residues (D+E)	11	11
# negatively charged residues (K+R)	3	11
# hydrophobic residues (A+C+H+I+L+M+ F+P+W+Y+V)	39	30
of which # surfaced exposed residues (> 2.5 Å^2 surface)	32	21

Supplementary Table 2. Data collection, phasing, and refinement statistics.

	USP36$^{81-461}$~Fubi-PA (PDB-ID: 8BS3)	USP36$^{81-424}$~Ub-PA (PDB-ID: 8BS9)
**Data collection**		
Beamline	SLS – PX2	SLS – PX2
Wavelength (Å)	0.9188	0.9999
Space group	$P\ 2_1\ 2_1\ 2_1$	$P\ 2_1$
Cell dimensions		
a, *b*, *c* (Å)	63.63, 75.89, 96.41	75.74, 65.31, 103.41
α, β, γ (°)	90, 90, 90	90, 93.27, 90
Observed reflections	124,723	322,016
Unique reflections	24,315	79,593
Resolution (Å)	59.63 – 2.20 (2.27 – 2.20)	45.24 – 1.90 (1.94 – 1.90)
R_{merge}	0.177 (0.759)	0.066 (0.999)
R_{meas}	0.196 (0.842)	0.076 (1.147)
$I/\sigma(I)$	6.0 (2.0)	11.8 (1.4)
$CC_{1/2}$	0.985 (0.505)	0.999 (0.604)
Completeness (%)	99.8 (99.8)	99.9 (100)
Redundancy	5.1 (5.3)	4.0 (4.2)
Wilson *B* (Å^{-2})	24.1	31.1
**Refinement**		
Resolution (Å)	2.20 Å	1.90 Å
No. reflections	45,243	152,803
R_{work} / R_{free} (%)	19.6 / 22.7	17.1 / 20.9
No. atoms	3,350	6,874
Protein	3,075	6,189
Ligands	6	14
Water	269	671
B factors (Å^{-2})	32.6	40.4
Protein (Å^{-2})	32.2	39.5
Ligands (Å^{-2})	26.8	38.7
Water (Å^{-2})	37.8	48.8
R.m.s.d.		
Bond lengths (Å)	0.002	0.010
Bond angles (°)	0.50	1.01
Ramachandran (favored / allowed / outlier) (%)	98.2 / 1.8 / 0	97.6 / 2.2 / 0.2
Clashscore	2.6	2.3
Rotamer outliers (%)	0.0	0.3
Copies / a.s.u.	1	2

Each dataset was collected from a single crystal. Values in parentheses are for the highest resolution shell. R.m.s.d., root mean square deviations.

Supplementary Table 3. Important motifs for Ubl-specific recognition.

	**Ubiquitin**	**Fubi**	**ISG15**
Ubl-specific patch (including Ile44-patch of Ubiquitin, termed „IBB-1" for ISG15[1])	Gln49<br>Arg42<br>Ile44<br>Val70	Pro47<br>Val40<br>Leu42<br>Ala68	Pro28<br>Trp121<br>Ser123<br>His149
Residue preceding terminal diGly motif[2]	Arg72	Leu72	Arg74
Ile36-patch	Ile36	Ile34	Val115
Phe4-patch	Phe4	Phe4	Leu83
E1-specificity residue[3]	Arg72	Arg70	Arg151

Supplementary Note

Chemical Synthesis

General methods

All chemicals and solvents are commercially available and were used without further purification. NMR spectra were recorded on a Bruker Avance III HD NanoBay (400 MHz) spectrometer. Mass spectra were recorded on an Agilent 1260 chromatography system coupled to an ESI-MS (single quadrupole, MSD).

Preparation of diethyl ((methylsulfonyl)methyl)phosphonate[4]

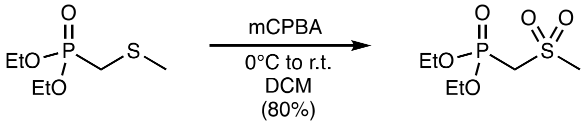

To a solution of diethyl ((methylthio)methyl)phosphonate (1.0 g, 5.05 mmol, 1.0 eq) in DCM (30 mL) at 0°C, mCPBA with 77% purity (2.5 g, 11.1 mmol, 2.0 eq) was added and the reaction was stirred at room temperature over night. The mixture was poured into a cold, saturated solution of K_2CO_3 and then extracted with DCM. The organic layers were combined, dried over $MgSO_4$, and concentrated to give the product as a white solid (926 mg, 80% yield), which was used without further purification. ESI-MS: $[M+H]^+$ cacld 231.0, found 231.0.

Preparation of *tert*-butyl (*E*)-(3-(methylsulfonyl)allyl)carbamate[5]

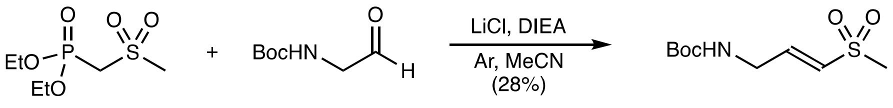

To a solution of diethyl ((methylsulfonyl)methyl)phosphonate (1.4 g, 6.1 mmol, 1.0 eq) in anhydrous MeCN, LiCl (344 mg, 8.11 mmol, 1.3 eq) and DIEA (1.18 mL, 6.8 mmol, 1.11 eq) were added under an argon atmosphere. Then *N*-Boc-2-aminoacetaldehyde (1.08 g, 6.78 mmol, 1.11 eq) in anhydrous MeCN was added dropwise. The reaction was monitored by TLC until the disappearance of diethyl ((methylsulfonyl)methyl)-

phosphonate. After 75 min, the solvent was evaporated, water (50 mL) was added, and it was extracted three times with ethyl acetate (50 mL each). The organic layers were combined, dried over $MgSO_4$, and purified via silica gel chromatography (PE:EA = 4:1 to 1:1, R_f = 0.22 at PE:EA= 1:1). Hexane was used for recrystallization to give the product as a white solid (400 mg, 28% yield). ^{1}H NMR (400 MHz, $CDCl_3$) δ 6.92 (dt, J = 15.1, 4.3 Hz, 1H), 6.50 (dt, J = 15.2, 1.9 Hz, 1H), 4.75 (s, 1H), 4.00 (s, 2H), 2.94 (s, 3H), 1.46 (s, 9H). ESI-MS: $[M+Na]^+$ cacld 258.1, found 258.1.

Preparation of (*E*)-3-(methylsulfonyl)prop-2-en-1-amine hydrochloride[5] (VS-HCl)

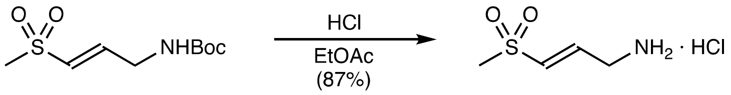

In a 250 mL double-neck round bottom flask, NaCl solid was stirred vigorously. Concentrated sulfuric acid was slowly added through a dropping funnel. The resulting HCl gas was bubbled into ethyl acetate to give a saturated HCl solution which was used directly for the next step. *tert*-Butyl (*E*)-(3-(methylsulfonyl)allyl)carbamate (396 mg, 1.68 mmol, 1.0 eq) was dissolved in ethyl acetate (2 mL), the flask was cooled on an ice bath, and then HCl in ethyl acetate solution (~10 mL) was added. The reaction was moved to room temperature and stirred overnight. The solvent was removed *in vacuo* to give the product as white solid (250 mg, 87% yield). ^{1}H NMR (400 MHz, DMSO-d_6) δ 8.47 (s, 3H), 7.02 (dt, J = 15.7, 1.6 Hz, 1H), 6.76 (dt, J = 15.4, 5.6 Hz, 1H), 3.72 (s, 2H), 3.04 (s, 3H). ESI-MS: $[3M+Na]^+$ cacld 428.1, found 428.4.

Supplementary References

1. Basters, A., Geurink, P.P., Rocker, A., Witting, K.F., Tadayon, R., Hess, S., Semrau, M.S., Storici, P., Ovaa, H., Knobeloch, K.P. & Fritz, G. Structural basis of the specificity of USP18 toward ISG15. *Nat Struct Mol Biol* **24**, 270-278 (2017).

2. Gjonaj, L., Sapmaz, A., Flierman, D., Janssen, G.M.C., van Veelen, P.A. & Ovaa, H. Development of a DUB-selective fluorogenic substrate. *Chem Sci* **10**, 10290-10296 (2019).

3. Schulman, B.A. & Harper, J.W. Ubiquitin-like protein activation by E1 enzymes: the apex for downstream signalling pathways. *Nat Rev Mol Cell Biol* **10**, 319-31 (2009).

4. Otrubova, K., Cravatt, B.F. & Boger, D.L. Design, synthesis, and characterization of alpha-ketoheterocycles that additionally target the cytosolic port Cys269 of fatty acid amide hydrolase. *J Med Chem* **57**, 1079-89 (2014).

5. Schwarz, J.B., Ly, C., Crawford, J., Roberts, T.C., Smith, P.A., Higuchi, R.I., Paraselli, P., Bergeron, P., Koehler, M.F.T. & Hu, H. Macrocyclic broad spectrum antibiotics. *WO2015179441A2* (2015).
