# Supplementary material for: Molecular basis for ubiquitin/Fubi cross-reactivity in USP16 and USP36
Source: Nat Chem Biol. 2023 Jul 13;19(11):1394–405. doi: 10.1038/s41589-023-01388-1 (PMC10611586; doi:10.1038/s41589-023-01388-1)
Supplement: Supplementary file 2 — Reporting Summary [file 41589_2023_1388_MOESM2_ESM.pdf]

nature portfolio

Corresponding author(s): Malte Gersch

Last updated by author(s): May 28, 2023

Reporting Summary

Nature Portfolio wishes to improve the reproducibility of the work that we publish. This form provides structure for consistency and transparency in reporting. For further information on Nature Portfolio policies, see our Editorial Policies and the Editorial Policy Checklist.

Statistics

For all statistical analyses, confirm that the following items are present in the figure legend, table legend, main text, or Methods section.

n/a	Confirmed	
☐	☒	The exact sample size (*n*) for each experimental group/condition, given as a discrete number and unit of measurement
☐	☒	A statement on whether measurements were taken from distinct samples or whether the same sample was measured repeatedly
☐	☒	The statistical test(s) used AND whether they are one- or two-sided<br>*Only common tests should be described solely by name; describe more complex techniques in the Methods section.*
☒	☐	A description of all covariates tested
☒	☐	A description of any assumptions or corrections, such as tests of normality and adjustment for multiple comparisons
☐	☒	A full description of the statistical parameters including central tendency (e.g. means) or other basic estimates (e.g. regression coefficient) AND variation (e.g. standard deviation) or associated estimates of uncertainty (e.g. confidence intervals)
☐	☒	For null hypothesis testing, the test statistic (e.g. *F*, *t*, *r*) with confidence intervals, effect sizes, degrees of freedom and *P* value noted<br>*Give P values as exact values whenever suitable.*
☒	☐	For Bayesian analysis, information on the choice of priors and Markov chain Monte Carlo settings
☒	☐	For hierarchical and complex designs, identification of the appropriate level for tests and full reporting of outcomes
☒	☐	Estimates of effect sizes (e.g. Cohen's *d*, Pearson's *r*), indicating how they were calculated

Our web collection on statistics for biologists contains articles on many of the points above.

Software and code

Policy information about availability of computer code

Data collection	Tecan SparkControl 2.3, Agilent Openlab 2.4.0.628, UNICORN 7.3.0.498
Data analysis	GraphPad Prism 9, ImageJ 1.53o, MaxQuant v.2.0.3.1, Perseus v.1.6.14.0, Microsoft Excel 16.68, ProMass 3.0 rev12, Phenix 1.19.2_4158, coot 0.9.6, Phaser 2.8.3, DIALS 3.6.2, Aimless 0.7.7, xia2 3.6.1, XDS 20220110, Image Lab 6.1, PyMOL 2.4.2, Jalview 2.11.2.6, ESPript 3

For manuscripts utilizing custom algorithms or software that are central to the research but not yet described in published literature, software must be made available to editors and reviewers. We strongly encourage code deposition in a community repository (e.g. GitHub). See the Nature Portfolio guidelines for submitting code & software for further information.

Data

Policy information about availability of data

All manuscripts must include a data availability statement. This statement should provide the following information, where applicable:

- Accession codes, unique identifiers, or web links for publicly available datasets
- A description of any restrictions on data availability
- For clinical datasets or third party data, please ensure that the statement adheres to our policy

Data have been deposited with the protein data bank (accession codes 8BS3 and 8BS9), and with ProteomeXchange (accession code PXD038455). Compound characterization data are provided in the Supplementary Information and uncropped gels and blots are included as Source Data Files. Numerical raw data are provided as a Source Data File. The study used the following protein structures previously deposited with the pdb: Accession codes 1UBQ, 2L7R, 1NBF. Protein

sequences for yeast ULP1 and human USP36 are available through Uniprot accession codes Q02724 and Q9P275, respectively.

Human research participants

Policy information about studies involving human research participants and Sex and Gender in Research.

Reporting on sex and gender	Human research participants were not involved in this study.
Population characteristics	Human research participants were not involved in this study.
Recruitment	Human research participants were not involved in this study.
Ethics oversight	Human research participants were not involved in this study.

Note that full information on the approval of the study protocol must also be provided in the manuscript.

Field-specific reporting

Please select the one below that is the best fit for your research. If you are not sure, read the appropriate sections before making your selection.

☒ Life sciences ☐ Behavioural & social sciences ☐ Ecological, evolutionary & environmental sciences

For a reference copy of the document with all sections, see nature.com/documents/nr-reporting-summary-flat.pdf

Life sciences study design

All studies must disclose on these points even when the disclosure is negative.

Sample size	For quantitative experiments, a sample size of at least 2 (DUB profiler screen), typically 3 to 6 independent experiments was chosen in line with what is the standard of the field in the molecular biosciences. For non-quantitative experiments, a sample size of at least 2, typically 3-4 independent experiments was chosen in line with what is the standard of the field in the molecular biosciences.
Data exclusions	Data exclusion occurred in the processing of crystallographic data as implemented in the respective software (e.g. in Phenix.Refine during the scaling of input intensities and subsequent outlier rejection according to expected intensity statistics). These processes took place completely automated as is the default in these programs and without any customization or user input.
Replication	All observations were made in at least two independent experiments, typically with technical triplicates, all with consistent results. The numbers of independent experiments is given in the "Statistics and Reproducibility" section and individual results are shown were possible.
Randomization	Randomization was not applicable as no experiments involving humans/animals were performed, and no included experiment were sensitive to the order of measurement/treatment
Blinding	Blinding was not carried out as no subjective analysis (e.g. scoring) was performed.

Reporting for specific materials, systems and methods

We require information from authors about some types of materials, experimental systems and methods used in many studies. Here, indicate whether each material, system or method listed is relevant to your study. If you are not sure if a list item applies to your research, read the appropriate section before selecting a response.

Materials & experimental systems

n/a	Involved in the study	
☐	☒	Antibodies
☐	☒	Eukaryotic cell lines
☒	☐	Palaeontology and archaeology
☒	☐	Animals and other organisms
☒	☐	Clinical data
☒	☐	Dual use research of concern

Methods

n/a	Involved in the study	
☒	☐	ChIP-seq
☒	☐	Flow cytometry
☒	☐	MRI-based neuroimaging

Antibodies

Antibodies used	Anti-FAU (proteintech, 13581-1-AP); Anti-Flag M2 (Sigma, F3165); Anti-HA (BioLegend, 16B12); Anti-GAPDH (Thermo Fisher, AM4300); Anti-USP7 (abcam, ab190183); Anti-USP16 (Biomol, A301-614A-T); Anti-USP36 (Biomol, A300-940A-T); Anti-mouse coupled to HRP (Sigma, NXA931); Anti-rabbit coupled to HRP (Sigma, GENA934)
Validation	Antibodies are validated for the application of Western Blotting on human proteomes per statements on the manufacturers' websites. Antibodies for USP7, USP16 and USP36 were further validated by RNA interference.<br><br>Validation statements of primary antibodies as per manufacturers' websites:<br>"13581-1-AP targets FAU in WB, IHC, ELISA applications and shows reactivity with human, mouse, rat samples."<br>"Monoclonal ANTI-FLAG® M2 antibody produced in mouse has been used in: immunoblotting, immunoprecipitation"<br>"Additional tested and reported applications of the 16B12 clone for the relevant formats include: western blot (WB)"<br>"AM4300: Applications: Western Blot (WB), Species Reactivity: Amphibian, Dog, Chicken, Fish, Human, Mouse, Non-human primate, Rabbit, Rat"<br>"ab190183: Rabbit polyclonal to HAUSP / USP7, Suitable for: WB, ICC/IF, Reacts with: Mouse, Rat, Chicken, Human, Xenopus laevis"<br>"A301-614A-T: Application: WB, IP, Antibody Type: Polyclonal, Species reactivity: human (Expected: bovine)"<br>"A300-940A-T: Application: WB, IP, IHC, Antibody Type: Polyclonal, Species reactivity: human (Expected: dog, horse, orangutan, rhesus monkey, gorilla, chimpanzee, white-tufted-ear marmoset, crab-eating macaque, little brown bat, northern white-cheeked gibbon)"

Eukaryotic cell lines

Policy information about cell lines and Sex and Gender in Research

Cell line source(s)	HeLa, HEK293, and MCF7 cells were purchased from the DSMZ repository (DSMZ no: ACC 57, ACC 305, ACC 115, respectively)
Authentication	Cells were used without authentication.
Mycoplasma contamination	Cells were tested for mycoplasma contamination with a negative result.
Commonly misidentified lines<br>(See ICLAC register)	No commonly misidentified lines were used in this study.
