# Supplementary material for: Molecular basis for ubiquitin/Fubi cross-reactivity in USP16 and USP36
Source: Nat Chem Biol. 2023 Jul 13;19(11):1394–405. doi: 10.1038/s41589-023-01388-1 (PMC10611586; doi:10.1038/s41589-023-01388-1)

Uncropped blots and gels for Figure 1

Figure 1d

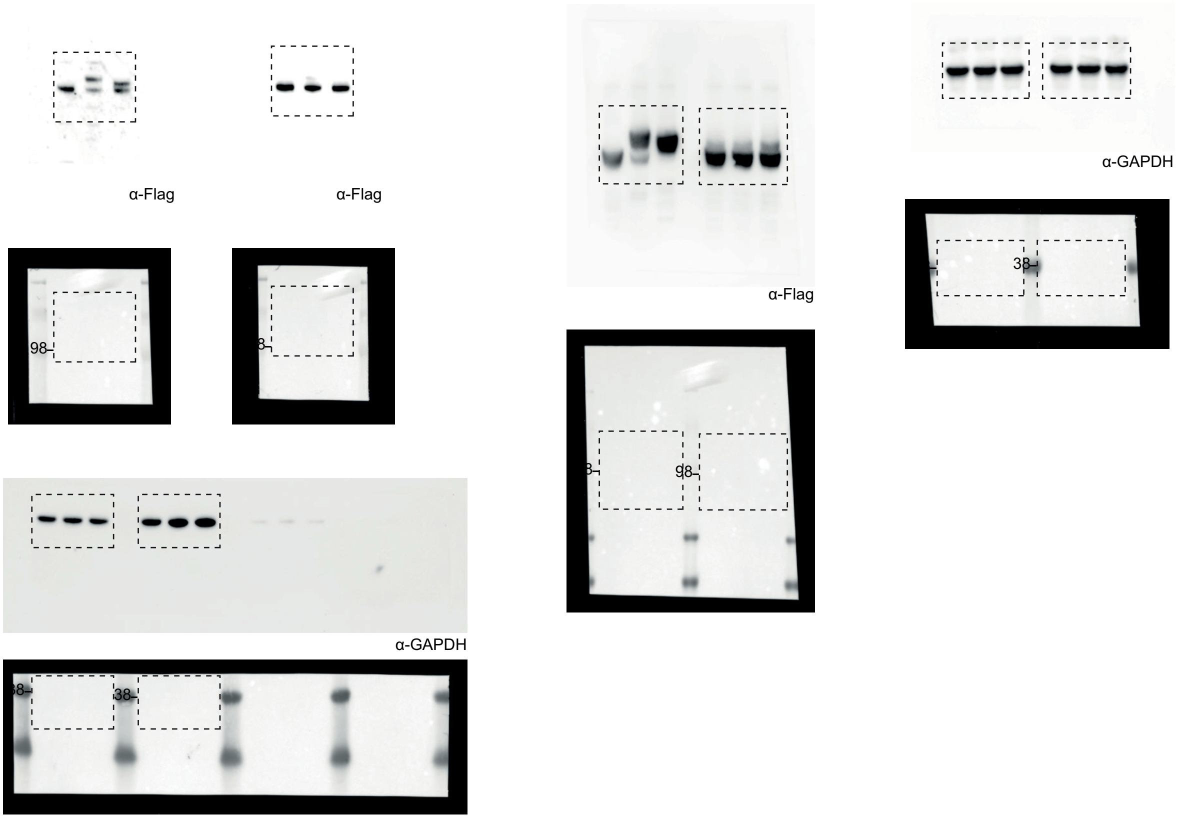

Uncropped blots and gels for Figure 1

Figure 1e

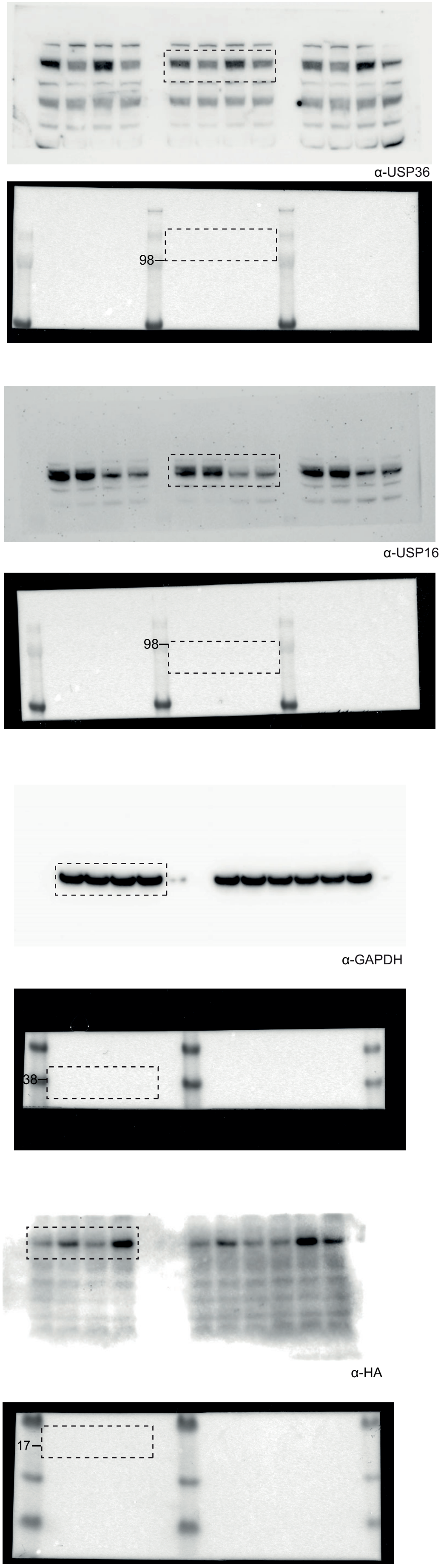

Figure 1g

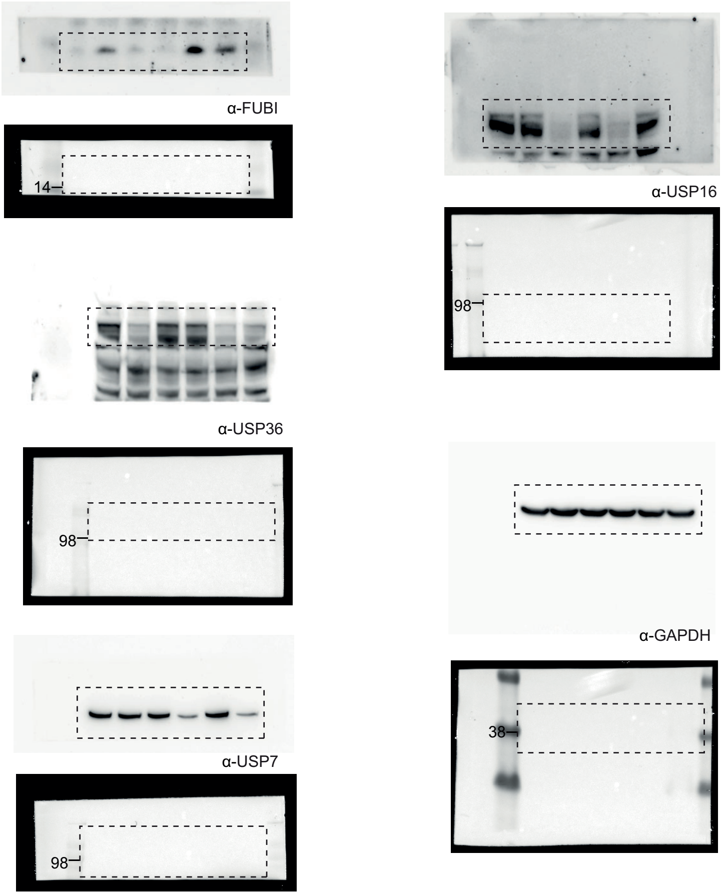

Supplement: Supplementary file 3 — Unprocessed western blots and/or gels. [file 41589_2023_1388_MOESM3_ESM.pdf]
