# Supplementary figures and images for: Molecular basis for ubiquitin/Fubi cross-reactivity in USP16 and USP36
Source: Nat Chem Biol. 2023 Jul 13;19(11):1394–405. doi: 10.1038/s41589-023-01388-1 (PMC10611586; doi:10.1038/s41589-023-01388-1)

## Uncropped blots and gels for Figure 3

Figure 3b

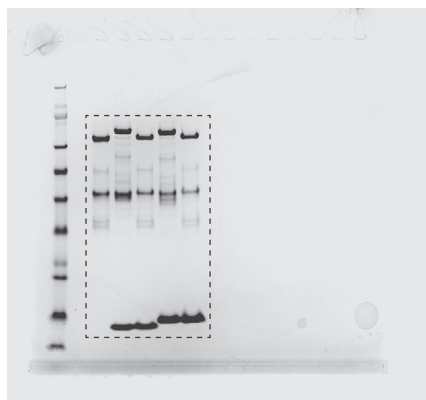

Figure 3c    Figure 3e

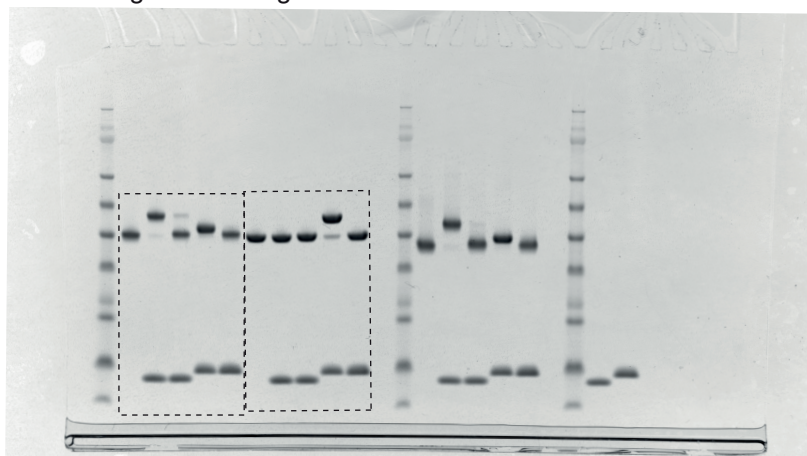

Supplement: Supplementary file 4 — Unprocessed western blots and/or gels. [file 41589_2023_1388_MOESM4_ESM.pdf]

Uncropped blots and gels for Figure 5

Figure 5i

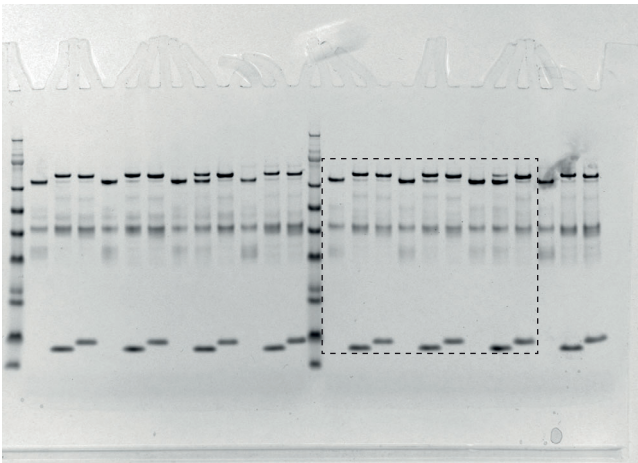

Figure 5j

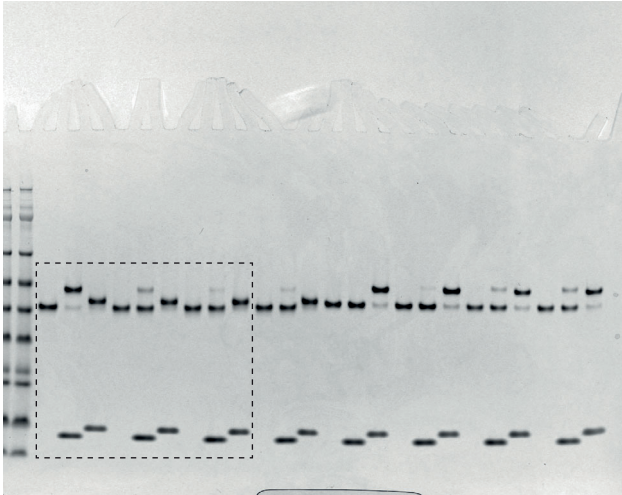

Figure 5k

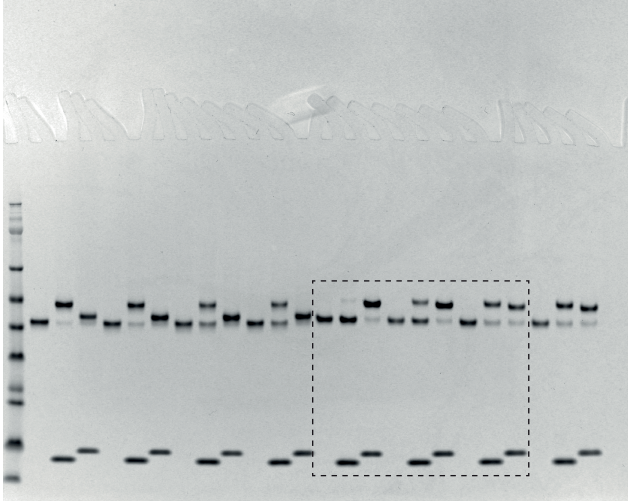

Supplement: Supplementary file 5 — Unprocessed western blots and/or gels. [file 41589_2023_1388_MOESM5_ESM.pdf]

Uncropped blots and gels for Figure 6

Figure 6b

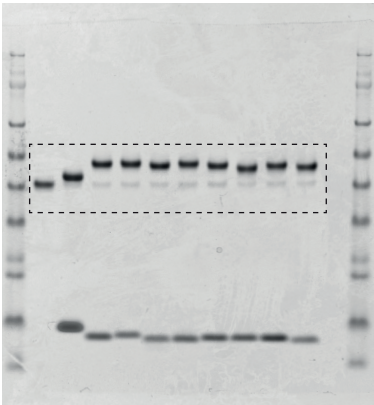

Figure 6c

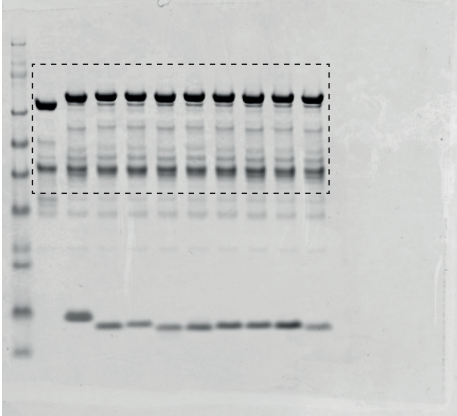

Figure 6d

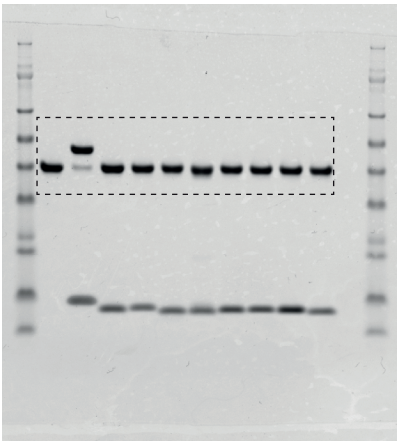

Figure 6e

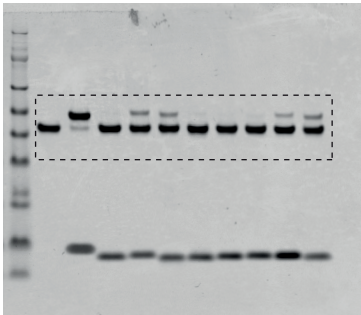

Figure 6f

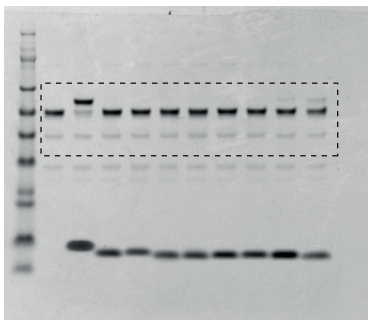

Supplement: Supplementary file 6 — Unprocessed western blots and/or gels. [file 41589_2023_1388_MOESM6_ESM.pdf]

## Uncropped blots and gels for Extended Data Figure 4

Extended Data Figure 4b

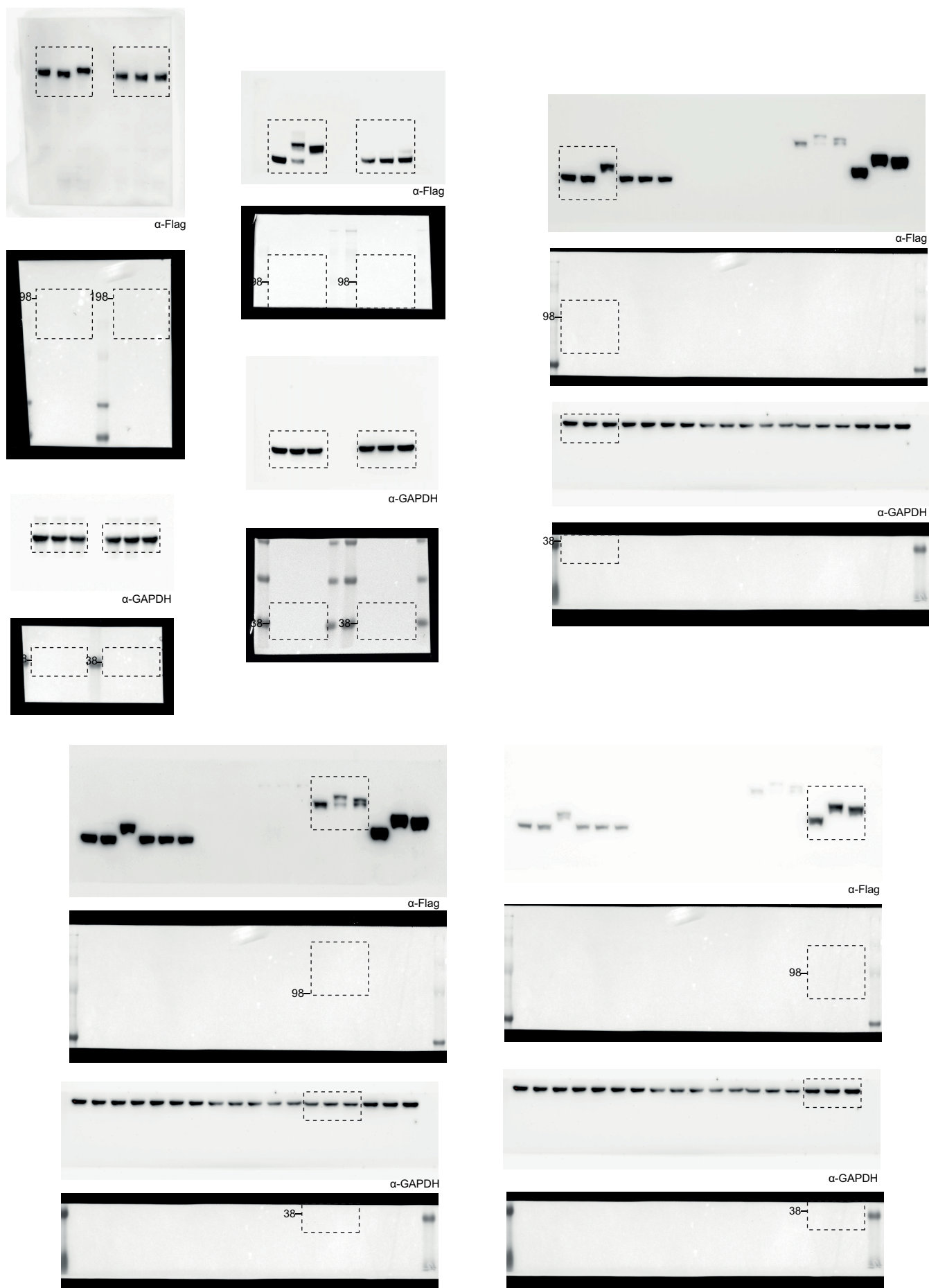

Supplement: Supplementary file 8 — Unprocessed western blots and/or gels. [file 41589_2023_1388_MOESM8_ESM.pdf]

Uncropped blots and gels for Extended Data Figure 5

Extended Data Figure 5c

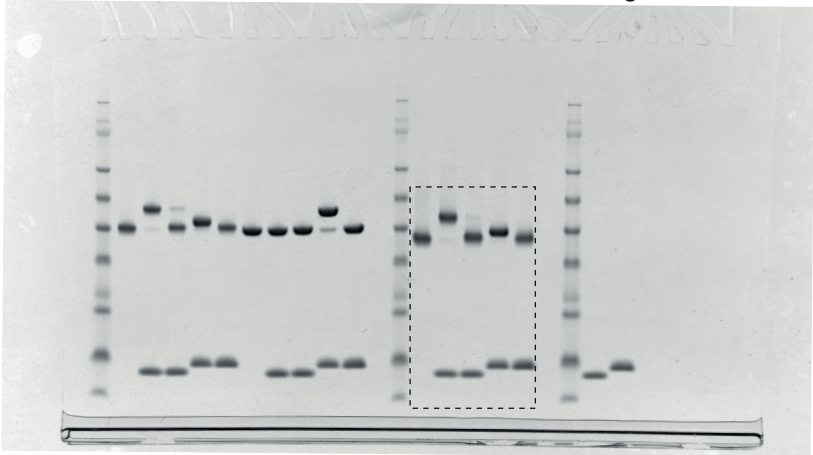

Supplement: Supplementary file 9 — Unprocessed western blots and/or gels. [file 41589_2023_1388_MOESM9_ESM.pdf]

Uncropped blots and gels for Extended Data Figure 9

Extended Data Figure 9a-b

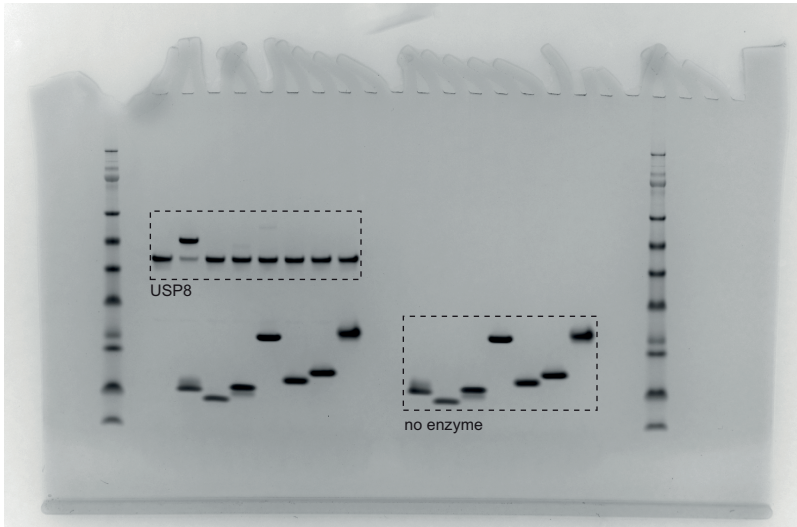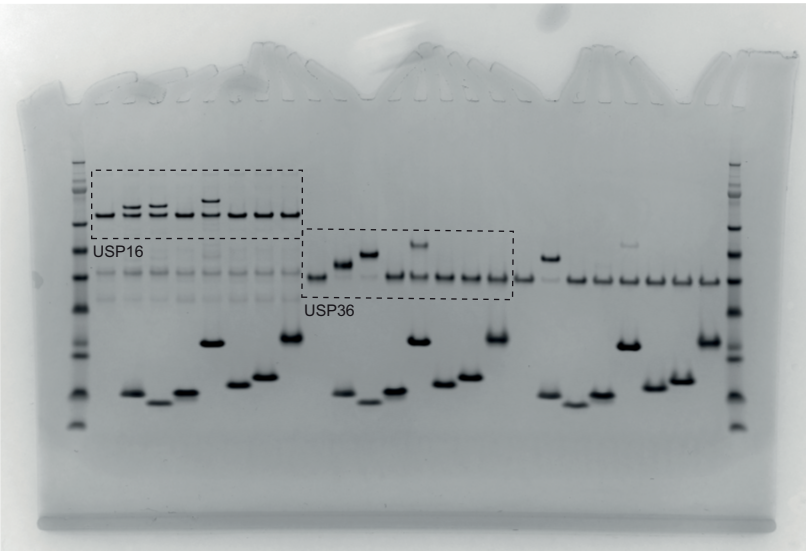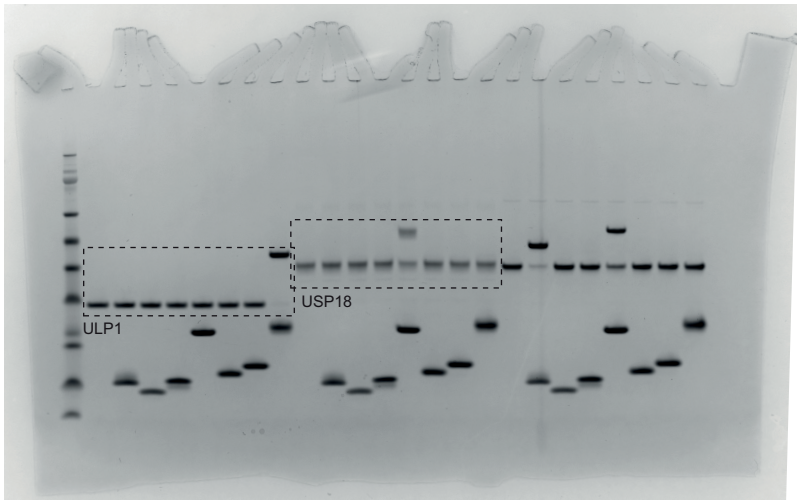

Supplement: Supplementary file 10 — Unprocessed western blots and/or gels. [file 41589_2023_1388_MOESM10_ESM.pdf]

Uncropped blots and gels for Extended Data Figure 10

Extended Data Figure 10c-e

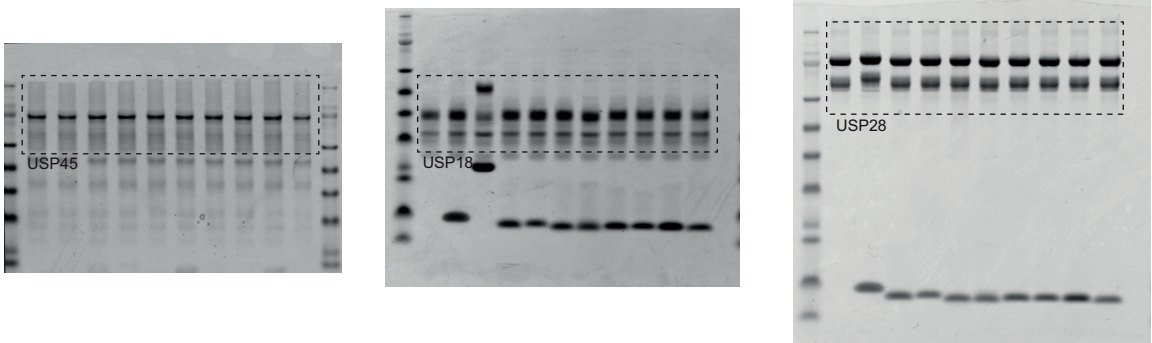

Supplement: Supplementary file 11 — Unprocessed western blots and/or gels. [file 41589_2023_1388_MOESM11_ESM.pdf]
